# Weighted gene coexpression network analysis-based identification of key modules and hub genes associated with drought sensitivity in rice

**DOI:** 10.1186/s12870-020-02705-9

**Published:** 2020-10-20

**Authors:** Baiyang Yu, Jianbin Liu, Di Wu, Ying Liu, Weijian Cen, Shaokui Wang, Rongbai Li, Jijing Luo

**Affiliations:** 1 College of Life Science and Technology (State Key Laboratory for Conservation and Utilization of Subtropical Agro-bioresources), Guangxi University, Nanning, 530004 China; 2 Agriculture College, South China Agricultural University, Guangzhou, 510642 China; 3 Agriculture College, Guangxi University, Nanning, 530004 China

**Keywords:** Rice, Transcriptomic profiling, Drought-sensitive phenotype, WGCNA, Photosynthesis inhibition, H_2_O_2_/MDA accumulation, DEGs encoding WRKYs/PR proteins

## Abstract

**Background:**

Drought stress is an adverse factor with deleterious effects on several aspects of rice growth. However, the mechanism underlying drought resistance in rice remains unclear. To understand the molecular mechanism of the drought response in rice, drought-sensitive CSSL (Chromosome Single-substitution Segment Line) PY6 was used to map QTLs of sensitive phenotypes and to reveal the impact of the QTLs on transcriptional profiling.

**Results:**

The QTL *dss-1* was mapped onto the short arm of chromosome 1 of rice. According to transcriptomic analysis, the identified differentially expressed genes (DEGs) exhibited a downregulated pattern and were mainly enriched in photosynthesis-related GO terms, indicating that photosynthesis was greatly inhibited under drought. Further, according to weighted gene coexpression network analysis (WGCNA), specific gene modules (designating a group of genes with a similar expression pattern) were strongly correlated with H_2_O_2_ (4 modules) and MDA (3 modules), respectively. Likewise, GO analysis revealed that the photosynthesis-related GO terms were consistently overrepresented in H_2_O_2_-correlated modules. Functional annotation of the differentially expressed hub genes (DEHGs) in the H_2_O_2_ and MDA-correlated modules revealed cross-talk between abiotic and biotic stress responses for these genes, which were annotated as encoding WRKYs and PR family proteins, were notably differentially expressed between PY6 and PR403.

**Conclusions:**

We speculated that drought-induced photosynthetic inhibition leads to H_2_O_2_ and MDA accumulation, which can then trigger the reprogramming of the rice transcriptome, including the hub genes involved in ROS scavenging, to prevent oxidative stress damage. Our results shed light on and provide deep insight into the drought resistance mechanism in rice.

**Supplementary information:**

**Supplementary information** accompanies this paper at 10.1186/s12870-020-02705-9.

## Background

Abiotic stresses such as drought, chilling, heat, and salinity are widespread factors with deleterious effects on several aspects of plants, including changes in metabolism, growth, and development, in extreme cases leading to plant death. However, as sessile organisms, higher plant usually adopt the ‘overcome’ strategy upon encountering any extreme environmental stresses; this strategy, in contrast to that of animals, is preferentially to opt for the tolerance or avoidance of unfavorable circumstances. In general, various tolerance/avoidance mechanisms [1], such as regulation of endogenous hormone signals [2], increases in wax deposition in the cuticle [3], and stomatal regulation [4], are employed in response to abiotic stresses.

Rice (*Oryza sativa* L.) is a staple food crop worldwide. Drought has been one of the major abiotic constraints of rice production with increasing frequency of global water shortages. Two important strategies, dehydration tolerance and avoidance, are employed by plants to cope with drought stress. Dehydration avoidance depends on the development of a large and deep root system to uptake water from soil and reduce water loss via the modulation of stomatal opening. Dehydration tolerance mainly enhances drought resistance via complex mechanisms that occur during later stages of drought stress, such as antioxidation processes, ABA signal transduction, osmotic adjustment, and cell membrane protection [5, 6]. A previous reports showed that two genotypes of Persian Walnuts (*Juglans regia* L.), Chandler’ and ‘Panegine20, exhibit high osmotic stress tolerance during seed germination [7]. The further study revealed that the strong accumulation of sugars and proline were detected in radicle and plumule of drought tolerant walnut varieties during germination, suggesting that sugars and proline are involved in the osmotic adjustment of plants in response to drought stress [8].

A large number of QTLs for root growth [9], osmotic pressure regulation [10], ROS regulation, and cuticular wax accumulation, which contribute to enhanced drought resistance, have been mapped to different locations of the rice genome via map-based cloning [11–13]. *DRO1*, one of the genes that has been well characterized, is involved in the regulation of rice root development under drought stress. *DRO1* regulates cell elongation in the root tip, which causes downward bending of the roots to avoid water deficit by increasing deep rooting [13]. *DST* was identified as a negative regulator of H_2_O_2_ accumulation in guard cells and functions in the modulation of stomatal opening. Loss of *DST* function increases stomatal closure, resulting in enhanced drought tolerance in rice [12]. In addition, *DS8* participates in the synthesis of plant cuticular wax and in the regulation of stomatal movement in response to drought stress [11]. Studies of these genes indicated that intricate mechanisms are employed by rice plants in the adaptation to drought stress. On the contrary, some drought stress-related QTLs were mapped through investigation of the drought sensitive traits. For example, 4 QTLs that contribute to increased transpiration rate under high vapour pressure deficit conditions were identified using F_7_ recombinant inbred lines of pearl millet [*Pennisetum glaucum (L.)* R. Br.]. All of these alleles were derived from drought-sensitive parent ICMB 841 [14]. Therefore, drought sensitivity is also an alternative trait for QTL identification in the study of drought tolerance mechanisms. In addition, an association analysis among phenotypic, genotypic, and environmental variables using single nucleotide polymorphisms (SNPs) was performed to identify the loci that are associated with drought tolerance in Persian walnut. Among candidate genes for the identified loci, majority of which are involved in ABA signaling, stomatal regulation, signal transduction, antioxidant defense, osmotic adjustment, and leaf growth and development [15].

Chloroplasts are organelles that perform photosynthesis in plant cells. As a key process for the responsive regulation, when rice plants are subjected to abiotic stresses, photosynthetic activity is greatly inhibited [16]. The inhibition of photosynthesis reduces the utilization of absorbed light energy, resulting in the generation of reactive oxygen species (ROS), including singlet oxygen, H_2_O_2_, and superoxide anion in chloroplasts, which in turn can cause photodamage to photosystem proteins such as D1, a core component of photosystem II [17]. Plants have evolved a series of enzymatic and nonenzymatic antioxidant systems. The enzymatic system, which involves superoxide dismutase (SOD), catalase (CAT), and ascorbate peroxidase (APX), participates in scavenging ROS and preventing damage from oxidative stress [18]. SODs function in the dismutation of O^2−^ to H_2_O_2_ and O_2_; the resultant H_2_O_2_ is then catalyzed into H_2_O and O_2_ by the activity of CAT and APX [19]. The overaccumulation of ROS, causes severe injury to plant cells, even leading to programmed cell death. Therefore, ROS homeostasis plays a key role in plant drought resistance regulation [20, 21]. It has been reported that the levels of antioxidant activity significantly enhanced with the increasing of POD, APX, CAT, SOD, and LOX enzymes in the drought tolerant genotypes of Persian walnut (*Juglans regia* L.) [22, 23]. Moreover, emerging evidence has indicated strong correlations among antioxidant mechanisms, photosynthesis regulation, and drought tolerance [20, 24, 25]. Specifically, ROS can act as secondary messengers that are involved in the stress signaling response, photosynthetic regulation, stomatal movement, and the development of higher plant [26]. In addition, ABA induces the accumulation of H_2_O_2_ via the activation of NADPH oxidase, which is activated by SnRK2 phosphorylation, eventually resulting in stomatal closure. However, on the other hand, the accumulation of ABA induces the expression of *CAT*, suggesting that a certain relationship exists between ABA signaling and H_2_O_2_ balance [27–32].

Recent reports have suggested that WRKY transcription factors (TFs) are involved in the regulatory mechanism pathways of abiotic stresses, including those in response to drought and salinity [33, 34]. WRKY TFs constitute a plant-specific group of zinc finger transcription factors that are characterized by containing a conserved WRKY domain and that bind to a consensus *cis*-element W-box (TTGACT/C) [35]. The majority of the more than 100 WRKY members in rice are involved in the plant defense response in either a negative or positive manner [35–37]. Some WRKY members have important roles in abiotic stress responses; for example, *OsWRKY13* has been suggested to regulate the antagonistic cross-talk between drought and disease resistance pathways via repression of *SNAC1* and *WRKY45–1* [33, 34]. Moreover, pathogenesis-related (PR) proteins have been well characterized as a group of proteins that are induced not only by infection from pathogens, such as viruses, bacteria, and fungi, but also in response to abiotic stresses, including drought and salinity [38]. All PR proteins in both monocots and dicotyledons can be classified into 17 families [39]. PR3 and PR4 proteins have plant chitinase activity, and the PR5 family of proteins includes permatins, osmotins, zeanatins, and thaumatin-like proteins [40]. *PR10* genes belong to a multigene family in various plant species and exert RNase activity and ligand binding activity and are involved in posttranslational modification (phosphorylation) and phytohormone signaling in response to stress. The promoters of *PR10* genes harbor *cis*-acting elements for WRKY, bZIP, ERF, and MYB transcription factors, suggesting PR10 genes have pivotal roles in the stress response pathways [40].

Given the rapid development of next-generation sequencing technology, RNA-seq has been widely used to explore the transcriptomic profiling of plants in response to challenges with adverse environmental conditions. Recent studies have revealed that antioxidant processes, the ABA pathway, and photosynthesis are the target biological processes for plants in response to environmental changes at the transcription level [41, 42]. Intriguingly, the photosynthesis rate was found to decline under drought stress, especially in drought-sensitive genotypes, suggesting that the enhancement of antioxidant capacity favors maintaining a higher photosynthetic activity and thereby improves the drought resistance of plants [42]. Moreover, phytohormone signaling and Ca^2+^ signaling have also been highlighted in transcript profiling and have been revealed cross-talk among phytohormone signaling pathways [43, 44]. In addition, a large number of transcription factor families, such as WRKY, bZIP, and MYB families, were found to be involved in ABA-mediated signal transduction under abiotic stress [45, 46]. However, the molecular mechanism of drought tolerance/sensitivity still needs to be further investigated.

Weighted gene coexpression network analysis (WGCNA) is a new technique that can be used to identify potential gene modules with the highest connectivity among genes, and these modules are correlated with certain phenotypes among the gene expression data [47, 48]. WGCNA involves the construction of a coexpression network that can indicate correlations among genes across samples. Based on the coexpression relationships, genes with similar expression are grouped into the same module, thus suggesting that genes in the same module may have similar functions or possibly have common biological regulatory roles [49]. This method has been successfully applied in numerous study cases that have been used to identify hub genes and to determine the relationships between gene expression data and relevant phenotypes [50–55].

Here, we identified a drought-sensitive rice CSSL PY6, which carries the substitution segment derived from the drought-sensitive variety Lambayeque 1, by backcrossing into the background of the drought-tolerant variety PR403. The *dss-1* locus underlying the drought-sensitive phenotype of PY6 was harbored on the short arm of chromosome 1. To investigate the impact of *dss-1* on the transcriptional profiling of PY6, RNA-seq was performed to identify genes that were differentially expressed under drought stress treatment. Further, WGCNA was applied to identify hub genes from the modules that were strongly correlated with drought-induced physiological indicators. By using this approach, we aimed to elucidate the *dss-1*-mediated potential regulatory network that is related to drought stress tolerance in rice.

## Results

### Identification of the *dss-1* locus for drought-sensitive phenotypes in CSSLs

**Fig. 1:**
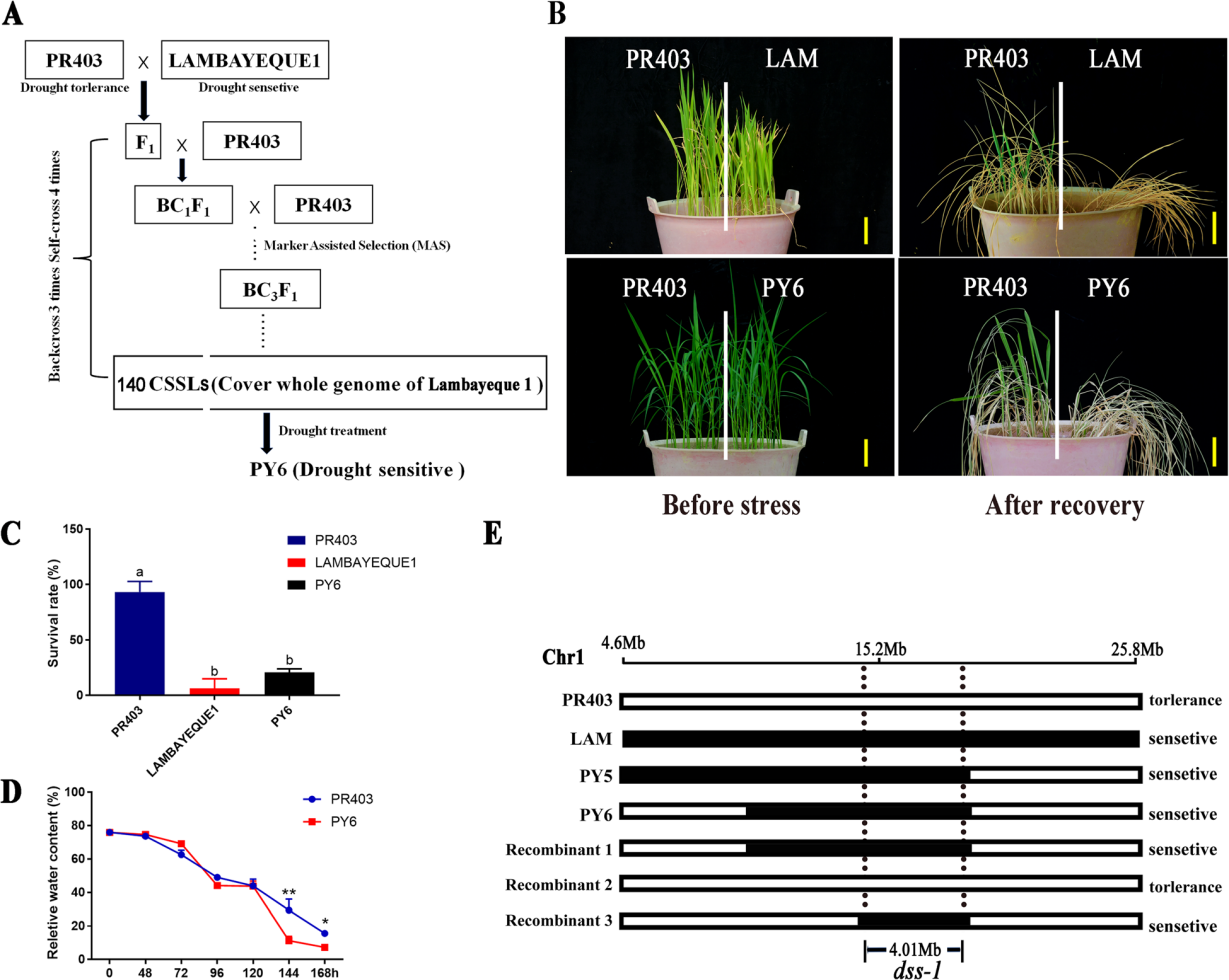
Characterization of a drought-sensitive CSSL. **a** Flowchart of the CSSL construction and drought-sensitive CSSL identification. **b** Drought stress phenotype of PR403, Lambayeque 1, and PY6. **c** Survival rate of PR403, Lambayeque 1, and PY6 after drought stress treatment. The different letters at the top of each column indicate statistically significant differences based on ANOVA with Tukey’s HSD test (*P* < 0.05). **d** Relative water content of leaves of PR403 and PY6 under drought stress treatment. The data are presented as the means ± SDs (*n* = 3; *, *P* < 0.05; **, *P* < 0.01; Student’s *t* test). **e** Fine mapping of the QTL *dss-1* underlying the drought-sensitive phenotype of PY6. Scale bar = 5 cm in (**b**)

A set of 140 rice CSSLs was constructed by introgressing the genomic segments of the drought-sensitive variety Lambayeque 1 into the genome of the drought-resistant variety PR403 through backcrossing (unpublished data; Fig. 1a). To elucidate the molecular regulatory mechanism underlying the drought-sensitive phenotype of Lambayeque 1, a drought stress treatment was applied to identify the major QTLs for drought-sensitive traits. PY6, one of the CSSLs, was highlighted for its drought-sensitive phenotype. After 10 days of drought treatment, the treated CSSLs were rewatered and allowed to recover for 3 days. In the screening, PY6, a CSSL, was identified as presenting a significant difference in survival rate (20.0%) compared with its recurrent parent PR403 (93.3%), whereas no significant difference was observed in the survival rate of PY6 and its donor parent Lambayeque 1 (Fig. 1b-c). During the drought stress treatment, the relative water content of the seedlings declined in PY6 and PR403 with decreasing soil moisture. However, starting at 120 h, a significant difference was observed between PY6 and PR403, and the former exhibited more severe dehydration at the later stage of the treatment, suggesting that drought stress disrupted the balance of water uptake and evaporation in PY6 (Fig. 1d). PY6 carries a 10-Mb chromosome segment derived from Lambayeque 1 on the short arm of chromosome 1. These results suggested that the segment from Lambayeque 1 may harbor a major QTL locus for the drought-sensitive phenotype of PY6. We designated this QTL as *dss-1*.

To reveal the genetic basis underlying the drought-sensitive phenotype afforded by the *dss-1* locus, PY6 was backcrossed with the recurrent parent PR403 to construct a mapping population. The F_1_ plants exhibited drought resistance, which was similar to that of the parental line PR403. Then, an F_2_ population was generated to determine the inherited features of *dss-1*. In a 151-individual F_2_ population, 111 members showed drought resistance, and 40 showed drought-sensitive traits (resistant:sensitive≈3:1, χ^2^ = 0.1788 < χ^2^_0.05_ = 3.84), indicating that drought-sensitive trait is controlled by a single recessive gene. In turn, the recombinants were screened for genetic linkage analysis, and *dss-1* was ultimately narrowed down to a 4.01-Mb interval (Fig. 1e).

### Drought induced H_2_O_2_ and MDA accumulation in the leaves of PY6

**Fig. 2:**
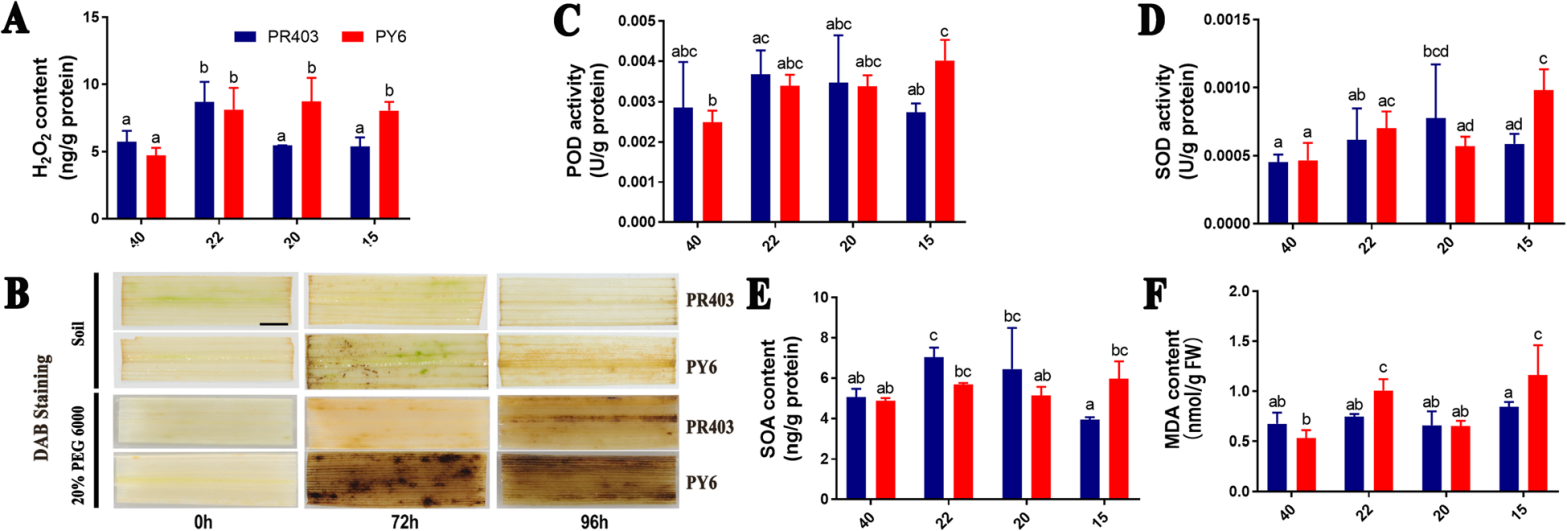
Comparison of ROS production and elimination in PY6 and PR403 under drought stress treatment. **a** H_2_O_2_ content. **b** DAB staining of leaf samples of PY6 and PR403. **c** POD activity. **d** SOD activity. **e** SOA content. **f** MDA content. The different letters at the top of each column in (**a**), (**c**), (**d**), (**e**), and (**f**) indicate statistically significant differences based on ANOVA with Tukey’s HSD test (*P* < 0.05). Scale bar = 0.5 cm in (**d**). FW in (**f**) represents fresh weight of leaf samples

Given that previous reports indicated that drought stress was known to inhibit photosynthetic activity in plants due to an imbalance between light capture and its utilization [56] and coupled with the considerable potential for increased accumulation of superoxide and hydrogen peroxide in chloroplasts [57], we examined the accumulation of ROS and the activity of its scavenging enzymes in the leaves of drought-treated PY6 plants. Leaf samples were harvested at 4 sampling points (Fig. S1). In contrast to PR403, the increased accumulation of H_2_O_2_ was detected in PY6 from points 40 to 15, whereas the enhanced content could be observed only at point 22 in PR403; therefore, the significantly increased accumulation of H_2_O_2_ was detected in PY6 at sampling points 22 and 15 compared with that of their respective controls (Fig. 2a). The marked accumulation of H_2_O_2_ in the leaves of PY6 was confirmed by DAB staining (Fig. 2b). Consistently, the activity of POD, an enzyme for H_2_O_2_ scavenging, was upregulated in response to the accumulation of H_2_O_2_ (Fig. 2c). However, the activity of APX and CAT, another two major enzymes for H_2_O_2_ scavenging, was not induced, and no significant difference was observed between either genotype during drought treatment (Fig. S2A, B). For the dynamic changes in superoxide activity, the content of SOA (superoxide anion) and the activity of its scavenging enzyme SOD at point 15 exhibited significant differences between the two genotypes with higher values in PY6. However, the content of SOA in the whole period of drought treatment was not significantly greater than that in the untreated control (point 40) (Fig. 2d, e; Fig. S2C). Moreover, the contents of MDA, a major reactive aldehyde resulting from the peroxidation of polyunsaturated fatty acid (PUFA) constituents of biological membranes and a compound that acts as an indicator of abiotic stress-induced biomembrane damage, were significantly higher in PY6 than in PR403 at points 22 and 15 (Fig. 2f).

Taken together, our results suggest that the drought-sensitive phenotype may be caused by the overaccumulation of ROS, especially H_2_O_2_, which could result in severe damage to plant cells and peroxidation of the cell membrane, thereby leading to the dehydration of rice plants under drought stress.

### Accumulation of ABA and stomatal modulation during drought stress treatment

As mentioned above, PY6 exhibited more severe dehydration at the later stage of treatment compared with the early stage, suggesting that PY6 either had a higher transpiration rate or lower water uptake ability when subjected to drought stress. ABA is a key regulator of plant stress responses and regulates a series of physiological processes, including tolerance to abiotic stresses and stomatal closure [58]. To determine the role of ABA-mediated stomatal movement in drought-induced severe dehydration in PY6, the ABA content and stomatal aperture status in drought-treated samples were examined. ABA accumulated during drought stress treatment, although no significant difference between PY6 and PR403 was observed at any sampling point (Fig. S2D). Scanning electron microscopy was used to examine the stomata status of leaf samples. Compared with that in the untreated control samples, the percentage of completely closed stomata in the drought-treated samples was significantly greater. However, PY6 and PR403 exhibited similar patterns in the ability to modulate their stomata in response to drought stress, and no significant difference in stomatal density was observed for either genotype (Fig. S2E). Therefore, these results suggested that severe dehydration in drought-treated PY6 do not result from a disruption of ABA-mediated stomatal transpiration regulation but, rather, may be caused by the limitation of water uptake or by other water loss pathways.

### Comparative transcriptional profiling of PY6 under drought stress

**Fig. 3:**
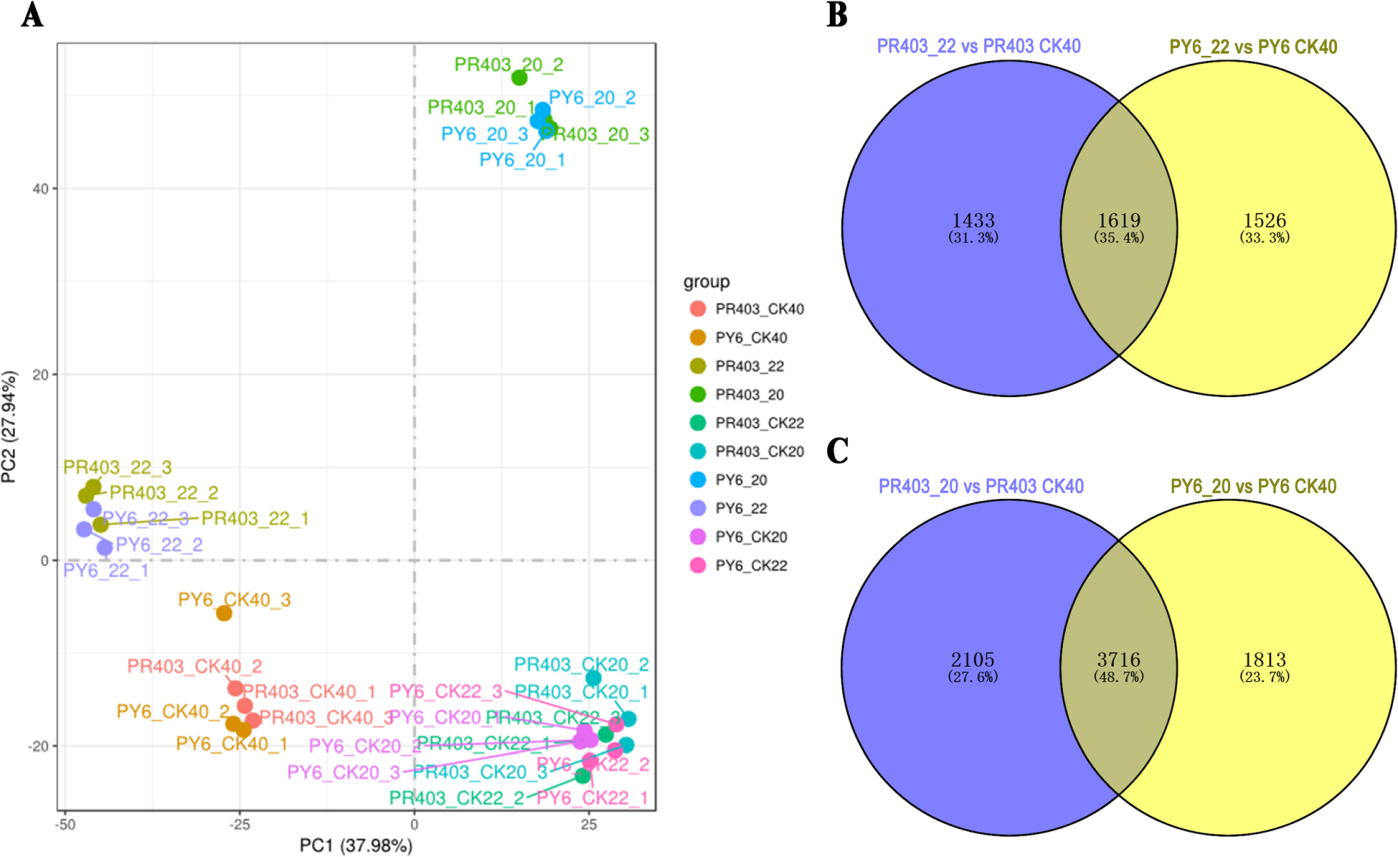
Global view of gene expression profiling under drought treatment. **a** Principal component analysis (PCA) of the RNA-seq data. **b-c** Venn diagrams showing the number of DEGs identified at two sampling points of PR403 and PY6. |log2FC| ≥1 and *padj* < 0.05 were used as thresholds for DEG identification. PR403/PY6_CK40, PR403/PY6_CK22, and PR403/PY6_CK20 in (**a-c**) represent untreated PR403/PY6 RNA-seq samples that were harvested at sampling point 40, 22, and 20, respectively. PR403/PY6_22 and PR403/PY6_20 in (**a-c**) represent drought treated PR403/PY6 RNA-seq samples that were harvested at sampling point 22 and 20, respectively

To further reveal the impact of *dss-1* on drought stress-induced H_2_O_2_ accumulation that may lead to the drought sensitivity of PY6, we investigated the transcriptional profiling of PY6 and PR403 in response to drought stress through RNA-seq. Principal component analysis (PCA) was initially performed based on all the transcriptional profiling data to detect the variations among the samples under the drought treatment (Fig. 3a). PC1 explained 37.98% of the total variation. The samples of CK40 and CK22/20 of both PY6 and PR403 were clearly separated by PC1, indicating the effect of the development imposed on the variation, while PC1 also separated the drought-treated samples of point 22 from those of point 20, indicating an effect of drought treatment on the transcriptomes of the samples. PC2 explained 27.94% of the total variation and clearly separated drought-treated samples from the parallel untreated samples (Fig. 3a). Moreover, both PY6 and PR403 samples from point 20 exhibited marked divergence from other sampling points, indicating that dramatic transcriptomic reprogramming occurred at this sampling point.

Drought-induced differentially expressed genes (DEGs) were identified by comparison with untreated controls (Table S2). It is worth noting that the development-dependent DEGs were detected in the same sampling points of well-watered samples under the same criteria, and they were excluded from the DEG set to avoid misinterpretation of drought-induced transcriptomic responses (Table S2–1 ~ − 8). Finally, at the two sampling points of PR403, 3052 DEGs, including 1135 upregulated and 1917 downregulated DEGs, were detected at point 22 (Table S2–9), while 5821 DEGs were detected at point 20, including 2383 upregulated and 3438 downregulated DEGs (Table S2–10). In PY6, 3145 (1748 upregulated/1397 downregulated) and 5529 (2655 upregulated/2874 downregulated) DEGs were detected at points 22 and 20, respectively (Table 1; Table S2–11, − 12). Among the resultant DEGs, Venn diagram analysis revealed that 1142 DEGs were common between PR403 and PY6 at point 22, while 3184 were common between PR403 and PY6 at point 20, which accounted for 38.4 and 48.7% of the total DEGs at the two sampling points, respectively (Fig. 3b-c; Table 1). These findings demonstrated that PY6 has a similar genetic background and a drought stress response as PR403.

**Table 1:** DEGs detected in PR403 and PY6 under drought stress treatment

Genotypes	Category of DEG	Sampling point	
		22	20
PR403	Up-regulated	1135	2383
	Down-regulated	1917	3438
	Total	3052	5821
PY6	Up-regulated	1748	2655
	Down-regulated	1397	2874
	Total	3145	5529
Common	Up-regulated	459	1202
	Down-regulated	683	1982
	Opposite-regulated	477	532
	Total	1619	3716

To verify the RNA-seq data quality, five genes were randomly selected for qRT-PCR. Similar trends of expression changes were observed between the qRT-PCR and RNA-seq data, indicating that the transcriptomic data were reliable for further analysis (Fig. S3A-E; Table S1).

### Gene ontology enrichment analysis

**Fig. 4:**
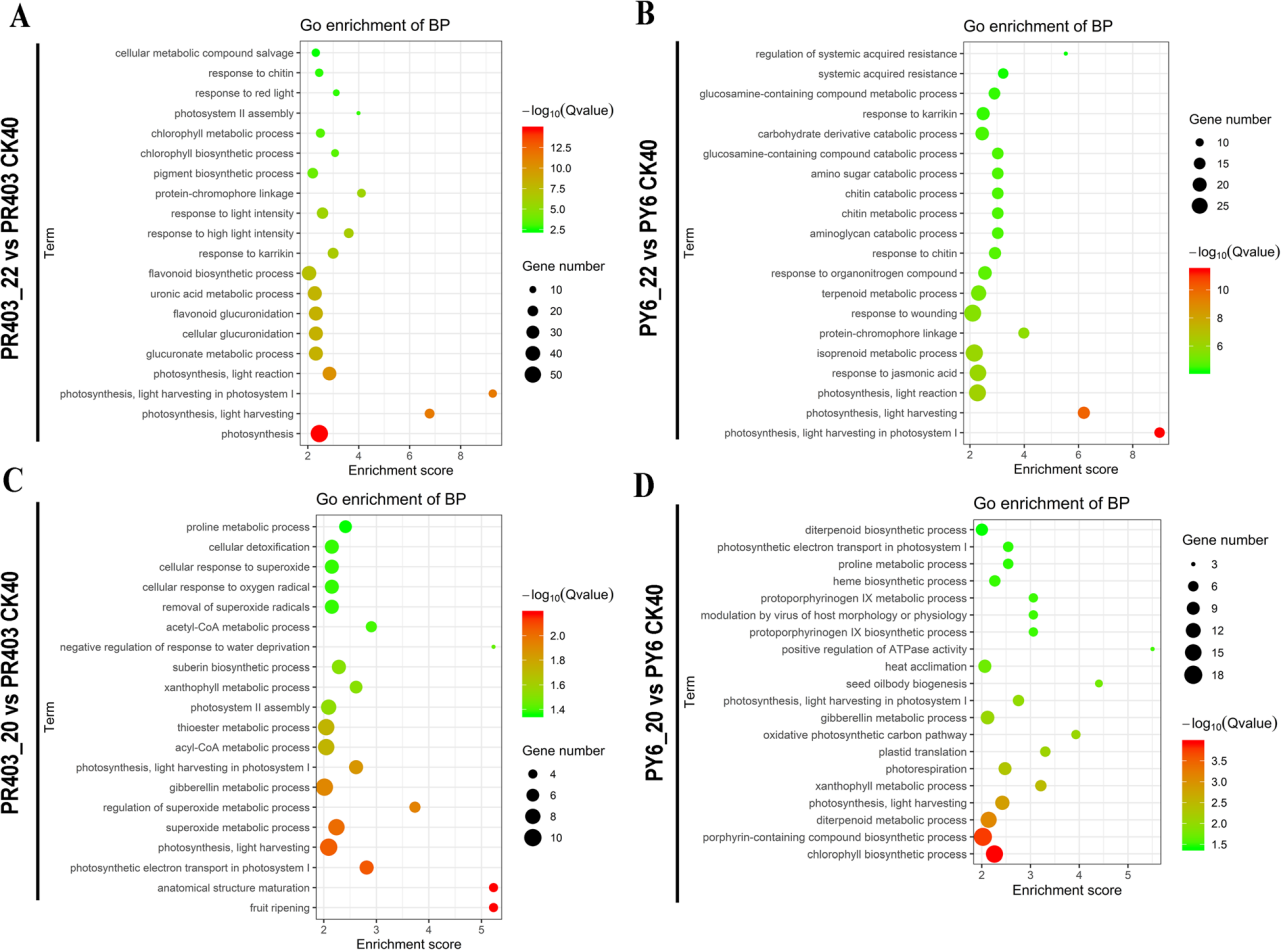
Top 20 biological process GO terms significantly overrepresented in the DEG GO enrichment analysis. **a** DEG-enriched GO terms at sampling point 22 of PR403. **b** DEG-enriched GO terms at sampling point 22 of PY6. **c** DEG-enriched GO terms at sampling point 20 of PR403. **d** DEG-enriched GO terms at sampling point 20 of PY6. Bubble size is proportional to the number of each GO-term, and the color represents the -log10 (*Qvalue*)

GO enrichment analysis was performed to cluster the identified DEGs into the functional categories of biological process (BP), cellular component (CC), and molecular function (MF) subgroups. The results showed that all the DEGs were enriched in the GO terms that were related to photosynthesis in the chloroplasts (Fig. 4a-d, *P* ≤ 0.01). For instance, in terms of the BP group, the GO terms involving photosynthesis (GO:0015979); photosynthesis, light harvesting (GO:0009765); photosynthesis, light harvesting in photosystem I (GO:0009768); photosynthesis, light reaction (GO:0019684); chlorophyll biosynthetic process (GO:0015995); and porphyrin-containing compound biosynthetic process (GO:0006779) were highly significantly overrepresented. The overrepresented GO terms, such as those involving chloroplast part (GO:0044434), the chloroplast envelope (GO:0009941), chloroplast thylakoid (GO:0009534), the chloroplast thylakoid membrane (GO:0009535), and the chloroplast thylakoid lumen (GO:0009543) in the CC category (Fig. S4A-D, *P* ≤ 0.01), and the overrepresented terms of the MF category, such as pigment binding (GO:0031409) and chlorophyll binding (GO:0016168) (Fig. S5A-D, *P* ≤ 0.01), supported the identified DEGs are related to photosynthetic modulation in response to drought stress. In addition, the superoxide metabolism-related GO terms superoxide metabolic process (GO:0006801) and regulation of superoxide metabolic process (GO:0090322) were highly enriched among the DEGs identified at point 20 of PR403, suggesting the involvement of ROS scavenging processes in the drought stress response (Fig. 4c, *P* ≤ 0.01). GO enrichment analysis suggested that photosynthetic modulation plays an important role in the regulatory network of rice plants in response to drought stress; therefore, chloroplasts are target organelles for the drought stress response.

### Construction of the coexpression network and identification of drought-induced hub genes

**Fig. 5:**
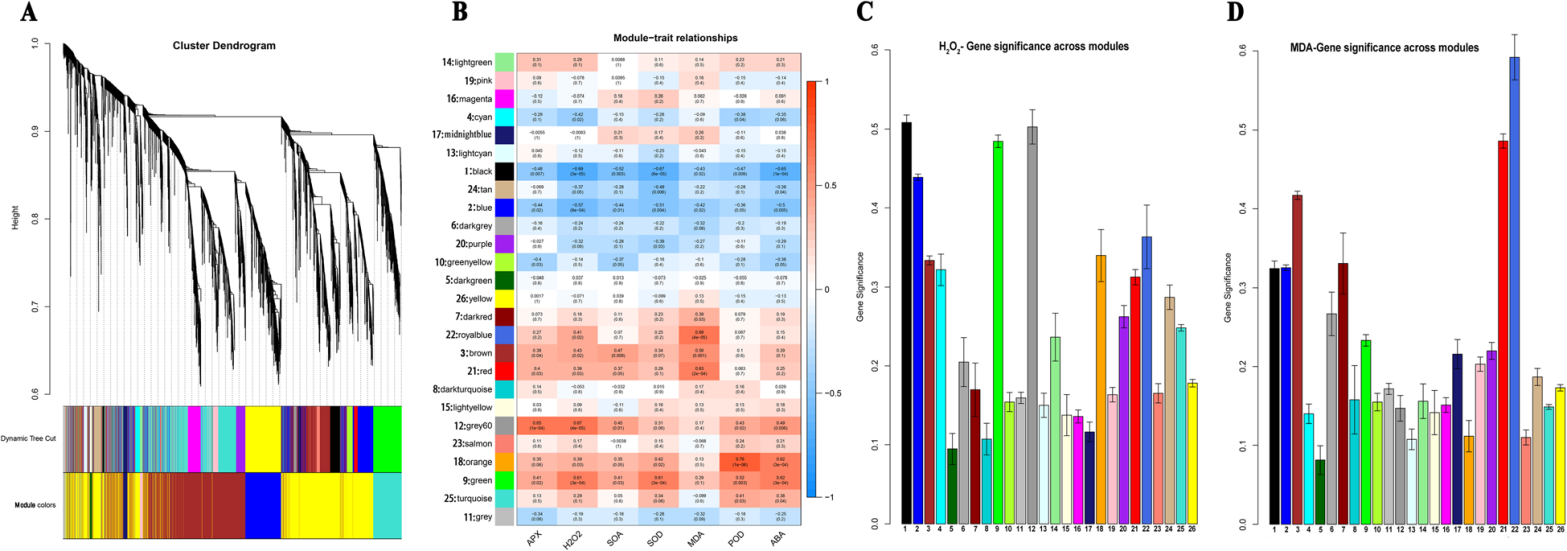
WGCNA coexpression network and module-trait correlation analysis. **a** Hierarchical cluster tree showing coexpression modules identified via the Dynamic Tree Cut method. Different modules are marked with different colors. Each leaf in the tree represents one gene. The major tree branches constitute 26 modules and are labeled with different colors. **b** Correlations of physiological indicators with WGCNA modules. Each row corresponds to a module and is labeled with the same color as that in a. The columns correspond to physiological indicators. The color of each cell indicates the correlation coefficient between the module and physiological indicator (the top number in the cell represents the correlation coefficient, and bottom one in parentheses represents the *P* value). **c** Correlations of H_2_O_2_ with WGCNA modules. **d** Correlations of H_2_O_2_ with WGCNA modules. The color bars with the numbers on the X-axis in (**c**) and (**d**) designate the modules corresponding to those with numbers and names shown on left side of (**b**)

To further investigate the impact of *dss-1*, a major QTL conferred PY6 drought sensitive phenotype, on the regulatory network in response to drought stress and to identify the specific genes that are strongly correlated with drought-induced physiological alterations in rice, we performed a WGCNA. After removing the genes with a low FPKM (FPKM< 1), a total of 23,178 genes were used to construct a scale-free coexpression network based on the soft-thresholding power of *β* = 12 (Fig. S6). According to the WGCNA results, the clusters with highly interconnected genes were defined as modules, and the genes in the same modules had high correlation coefficients. A total of 26 modules (coded with different colors to indicate different modules) were identified via the Dynamic Tree Cut method (core parameter: MEDissThres = 0.25) (Fig. 5a). With respect to the correlations of physiological traits with overrepresented modules, intriguing results were observed in the correlations. In general, the modules black, blue, and tan were negatively correlated with the majority of physiological traits, whereas the modules royalblue, brown, red, grey60, orange, and green were positively correlated with the traits, indicating that the genes clustered in a module have similar altered expression patterns in response to drought stress (Fig. 5b). Considering that significant differences in the contents of H_2_O_2_ and MDA were observed during drought stress, we focused on the correlations of H_2_O_2_ and MDA with gene modules and identified hub genes that were strongly correlated with the two drought-related physiological indicators (Fig. 2a, b, f; Fig. 5b, c, d). With the cut-off threshold of GS (Gene Significance) > 0.4, in all 26 of the modules, black (741 genes; R^2^ = -0.69, *P* = 3.0 × 10^− 5^), blue (4810 genes; R^2^ = -0.57, *P* = 9.0 × 10^− 4^), grey60 (148 genes; R^2^ = 0.67, *P* = 4.0 × 10^− 5^), and green (1099 genes; R^2^ = 0.61, *P* = 3.0 × 10^− 4^) were considered the modules that were strongly correlated with H_2_O_2_, and red (913 genes; R^2^ = 0.63, *P* = 2.0 × 10^− 4^), brown (3132 genes; R^2^ = 0.56, *P* = 1.0 × 10^− 3^), and royalblue (45 genes; R^2^ = 0.68, *P* = 4.0 × 10^− 5^) modules were considered strongly correlated with MDA (Fig. 5b, d). The genes in which the modules were clustered showed strong correlation with their respective modules (Fig. S7A, B). Furthermore, hub genes were identified based on the following cut-off thresholds: GS > 0.60 and MM (Module Membership) > 0.80 for the modules black, blue, green, and royalblue, and GS > 0.5 and MM > 0.8 for the modules grey60, red, and brown according to the threshold strengthening. After removing the genes with |log_2_FC| < 1, the expression of all the obtained hub genes was significantly differentially altered in PY6 and PR403 under drought stress and were highlighted in subsequent functional analyses (Fig. S8A-F; Table S3–1 ~ − 7). Hereafter, we designated these hub genes as DEHGs.

### Functional enrichment analysis of hub genes correlated with H_2_O_2_ accumulation

**Fig. 6:**
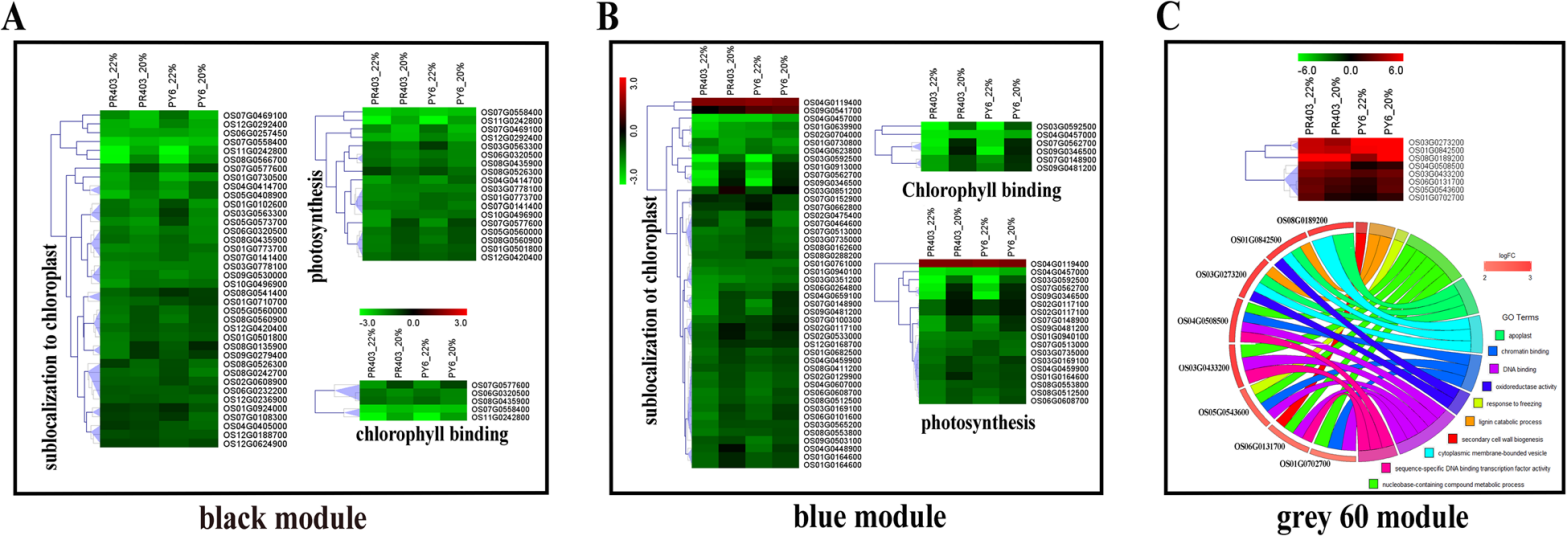
Heatmap and circular plot showing the altered expression and functional annotations of hub genes in the modules correlated with H_2_O_2_ accumulation. **a-b** Expression patterns of hub genes of the modules black and blue showing that they are related to photosynthesis of rice plants. **c** Expression patterns and GO enrichment of hub genes of the module grey60. Different colors represent hub gene-enriched corresponding GO terms. All the data used in the analysis were subjected to log2 transformation

GO enrichment analysis was performed to functionally cluster the hub genes in the modules that were strongly correlated with H_2_O_2_. A total of 115 and 204 DEHGs were identified in the modules black and blue, respectively (Table S3–1, − 2). In line with the abovementioned results, these hub genes were highly enriched in the GO terms involving chloroplast (GO:0009507); the chloroplast envelope (GO:0009941); thylakoid (GO:0009579); chloroplast stroma (GO:0009570); chlorophyll binding (GO:0016168); photosynthesis (GO: GO:0015979); photosystem I (GO:0009522); photosynthesis, light harvesting (GO:0009765); and photosynthesis, light reaction (GO:0019684). These terms are related to photosynthesis (*Q*value ≤ 0.05) (Fig. S9A-B; Table S5–1, − 2). Interestingly, in agreement with previous reports [59], the expression of the majority of the DEHGs in the modules black and blue was downregulated without marked log_2_FC differences (|log_2_FC_PY6_-log_2_FC_PR403_| ≥ 1, *P* < 0.05) observed between PY6 and PR403 during drought treatment (Fig. 6a, b; Fig. S8A, B), implying that PY6 and PR403 have a common drought-responsive regulation and that their photosynthetic activities were inhibited in response to drought stress.

The module grey60 was enriched with 41 DEHGs (Table S3–3). The expression of most of these DEHGs was elevated under drought stress (Fig. S8C). Among these genes, eight showed differential expression with markedly different magnitudes at least at one sampling point between two genotypes, exhibiting a strong dynamic induction in response to drought treatment (|log_2_FC_PY6_-log_2_FC_PR403_| ≥ 1, *P* < 0.05) (Fig. 6c; Table 2). The expression of Os01G0842500 and Os03G0273200, both of which encodes proteins that are similar to laccase and both of which were enriched in GO terms involving the apoplast (GO:0048046)/lignin catabolic process (GO:0046274)/cytoplasmic membrane-bounded vesicle (GO:0016023)/oxidoreductase activity (GO:0016491), was consistently enhanced at two points of both PY6 and PR403 and showed higher magnitudes in PY6 (particularly at point 20) (Fig. 6c; Fig. S9C; Table 2; Table S5–3). Five genes (Os01G0702700, Os06G0131700, Os05G0543600, Os03G0433200, and Os04G0508500), which are annotated as transcription factors and mainly enriched in GO terms involving DNA binding (GO:0003677), sequence-specific DNA binding transcription factor activity (GO:0003700), and response to freezing (GO:0050826), were significantly consistently induced to a certain extent at point 22 and were downregulated at point 20 in PR403, whereas they were significantly induced only at point 20 in PY6, with the exception of Os03G0433200 (Fig. 6c; Table 2; Table S5–3). Os01G0702700, Os05G0543600, and Os04G0508500 are MYB family TFs, and Os06G0131700 is a NAC TF. A recent study suggested that Os06G0131700 (*OsSWN1*) is related to secondary cell wall formation [60], implying that secondary cell wall formation may play a certain role in drought stress resistance. In addition, Os08g0189200 is annotated as a gene encoding Germin-like protein 8–3 and was suggested to be involved in disease resistance [61]. A different upregulation pattern of this gene was observed in PY6 and PR403 in response to drought stress (Fig. 6c; Table 2).

**Table 2:** Differentially expressed hub genes identified in the module grey60

Gene_id	PR403_22^**a**^	PR403_20^**b**^	PY6_22	PY6_20	Description	GO enrichment
Os01G0842500	4.69^**c**^	4.40	5.85	**8.49**	Similar to Laccase (EC 1.10.3.2).	GO:0005507, GO:0009809, GO:0016722, GO:0046274, GO:0048046, GO:0052716, GO:0055114
Os03G0273200	4.65	3.70	6.79	**9.00**	Similar to Laccase (EC 1.10.3.2).	GO:0005507, GO:0009809, GO:0016722, GO:0046274, GO:0048046, GO:0052716, GO:0055114
Os01G0702700	**2.20**	0.81	0.41	1.16	Similar to Transcription factor MYB86 (Myb-related protein 86) (AtMYB86) (Myb homolog 4) (AtMyb4).	GO:0003677
Os05G0543600	**2.56**	1.51	0.79	2.28	Similar to Myb-related protein.	GO:0003677
Os04G0508500	**3.65**	**3.34**	0.50	1.18	MYB family transcription factor, putative, expressed.	GO:0003677
Os06G0131700	**2.38**	1.80	0.98	2.16	Similar to NAM protein.	GO:0003677, GO:0005634, GO:0006355
Os08G0189200	**7.90**	7.40	3.48	6.64	Germin-like protein 8–3, Disease resistance	GO:0005618, GO:0030145, GO:0033609, GO:0045735, GO:0046564, GO:0048046
Os03G0433200	3.04	2.62	1.94	2.63	SHR transcription factor, Regulation of the number of cortex cell layers in the root, Coordination of stomatal patterning	GO:0003700, GO:0005634, GO:0006355, GO:0008356, GO:0009956, GO:0043565, GO:0045930, GO:0048366, GO:0055072

Notes: ^a^ represents the samples collected at the 22% of soil moisture content; ^b^ represents the samples collected at the 20% of soil moisture content; ^c^ the value represents the log2 transformation of the fold change of the expression

A total of 96 hub genes were identified in the module green, including 36 downregulated and 60 upregulated genes in response to drought (Fig. S8D; Table S3–4). GO enrichment analysis revealed that the majority of the overrepresented GO terms were related to oxidation-reduction process (GO:0055114), response to salt stress (GO:0009651), carbohydrate metabolic process (GO:0005975), biosynthetic process (GO:0009058), and metabolic process (GO:0008152). These hub genes were sublocalized to distinct cellular components and have various molecular functions (Fig. S9D; Table S5–4). However, with the exception of iron-sulfur cluster binding (GO:0051536), no GO term was significantly overrepresented in our analysis (Table S5–4). It is worth noting that only 1 hub gene, OS04G0685300, which encodes a protein with harpin-induced 1 domain, showed significant differential expression with a greater upregulation magnitude at two points of PY6 (2.03/2.67) compared with those in PR403 (Table S3–4).

Taken together, the functional annotation of the hub genes identified in the H_2_O_2_-related modules suggested that both PY6 and PR403 experienced inhibition of drought-triggered photosynthetic activity, leading to the accumulation of H_2_O_2_ in the rice plants. The hub genes with highly differential expression identified in the module grey60 may be responsible for the accumulation of H_2_O_2_ in rice plants.

### Functional enrichment analysis of hub genes correlated with MDA accumulation

**Fig. 7:**
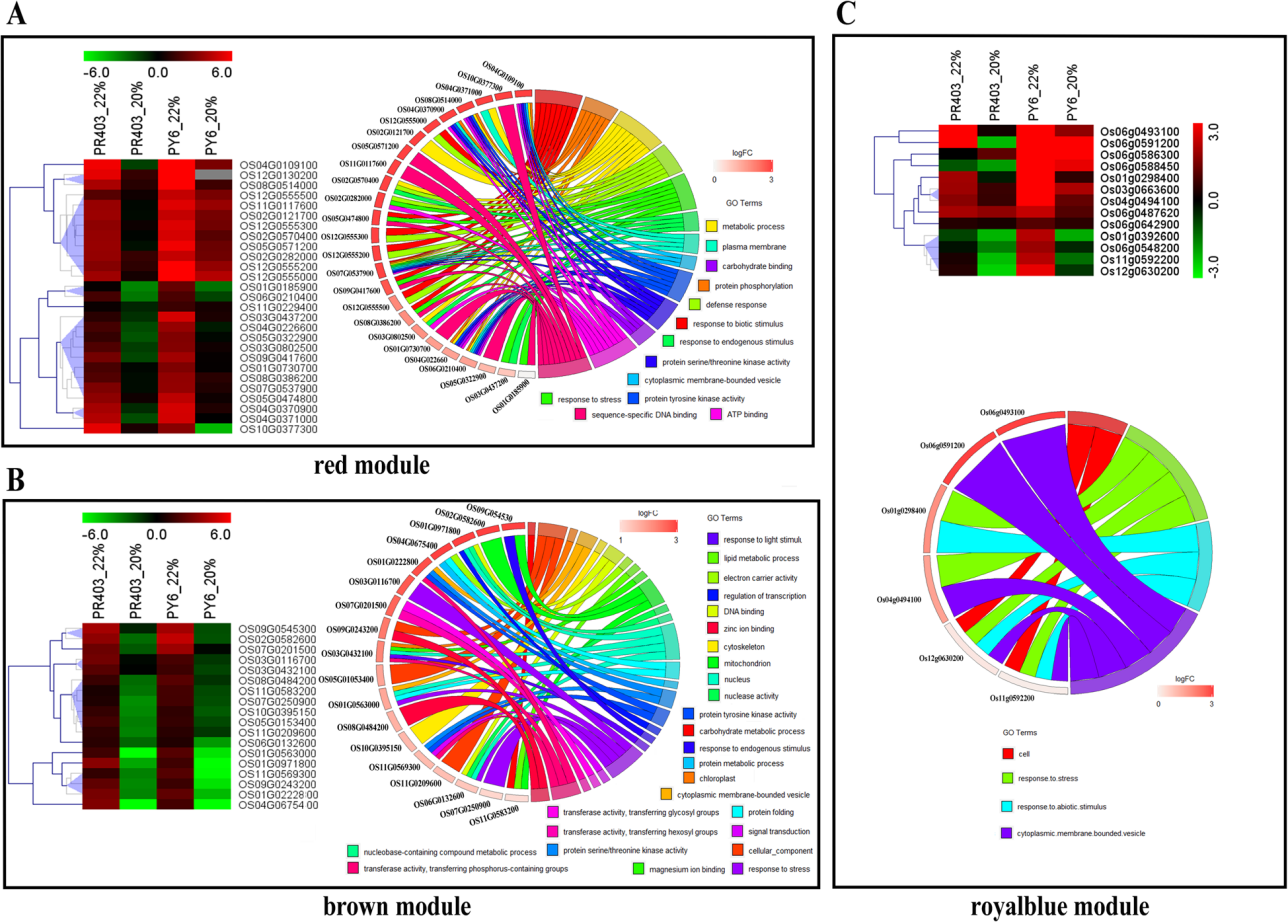
Heat map and circular plot showing the altered expression and functional annotations of hub genes in the modules correlated with MDA accumulation. **a** Expression patterns and GO enrichment of hub genes of the module red. **b** Expression patterns and GO enrichment of hub genes of the module brown. **c** Expression patterns and GO enrichment of hub genes of the module royalblue. All the data used in the analysis were subjected to log2 transformation. Different colors represent hub gene-enriched corresponding GO terms

MDA is an important indicator of membrane lipid peroxidation. The modules red, brown, and royalblue were strongly correlated with MDA under drought treatment; there were 303, 304, and 13 hub genes identified in these modules, respectively (Table S3–5 ~ − 7). For the module red, GO analysis revealed that the hub genes are involved in biotic stress and defense responses (Fig. S10A; Table S5–5). Among these hub genes, 29 showed differential expression at least at one sampling point between two genotypes (|log2FC_PY6_-log2FC_PR403_| ≥ 1, *P* < 0.05) (Fig. 7a; Table 3). Strikingly, most of them consistently showed similar expression patterns in both genotypes, with elevated transcripts at point 22, after which the expression levels then declined to levels that were comparable to or even lower than those of their respective untreated controls (Fig. S8E; Table 3). GO analysis revealed that these genes were involved in biotic stress responses; for example, the GO terms involving defense response (GO:0006952), response to biotic stimulus (GO:0009607), response to stress (GO:0006950), and response to endogenous stimulus (GO:0009719) were overrepresented in the results (Fig. 7a; Table S5–5). Of these genes, 8 were WRKY family TFs (Fig. 7a; Table 3). Specifically, *OsWRKY70* (Os05g0474800) and *OsWRKY76* (Os09g0417600) have been suggested to participate in ABA signaling and the biotic stress response [34, 62]. OS01g0185900 and OS05g0322900 exhibited relatively higher upregulated expression at point 22, whereas two other genes, OS05g0571200 and OS11G0117600, had relatively higher expression at point 20 of PY6 than at PR403 (Fig. 7a; Table 3). Considering the accumulation of MDA in PY6 and PR403, these results implied that four genes may be positively correlated with the overaccumulation of MDA and lead to the death of PY6 under drought stress. Moreover, *PR-10a* (OS12g0555300), which functions at the downstream of jasmonic acid pathway and is positively regulated by WRKY TFs in response to drought and high-salt stresses [38, 63], and three other PR-10 protein family hub genes (Os12g0555200, Os12g0555500, and Os12g0555000), which were annotated as genes encoding probenazole-inducible PBZ1 proteins and are involved in disease resistance, exhibited a higher magnitude of upregulated expression at two sampling points of PY6 compared with PR403 (Fig. 7a; Table 3). *BSR-d1/ZFP36* (Os03g0437200), which encodes a C_2_H_2_-type zinc finger protein, participates in ABA-OsMPK transduction, which results in the accumulation of H_2_O_2_ [64, 65]. Similarly, this gene also had a higher upregulated expression in PY6 than in PR403 (Table 3). In addition, the expression of OS02g0121700 and OS02g0570400 was dramatically induced in PY6 during the whole period of drought treatment, whereas these genes were significantly induced only at point 22 of PR403, after which their expression levels decreased to those of the untreated control (Fig. 7a; Table 3). These genes encode a terpenoid synthase domain-containing protein and *ent*-kaurene synthase 1A, which are involved in gibberellin (GA) biosynthesis, suggesting a role of GA in the drought stress response in rice. OS04g0109100, a gene annotated as concanavalin A-like lectin/glucanase, had a similar expression pattern in both genotypes.

**Table 3:** Differentially expressed hub genes identified in the module red

Gene_id	PR403_22^**a**^	PR403_20^**b**^	PY6_22	PY6_20	Description	GO enrichment
OS01G0730700	1.71^**c**^	−0.16	3.00	0.15	WRKY transcription factor 14 (WRKY14).	GO:0003700, GO:0006355, GO:0043565
OS05G0474800	3.36	0.38	4.46	0.36	WRKY transcription factor 70.	GO:0003700, GO:0006355, GO:0043565
OS09G0417600	2.76	−1.65	3.82	0.17	WRKY transcription factor 76.	GO:0003700, GO:0006355, GO:0009617, GO:0009620, GO:0009751, GO:0010200, GO:0043565
OS08G0386200	1.95	−0.28	3.46	0.99	WRKY transcription factor 69.	GO:0003700, GO:0006355, GO:0043565
OS01G0185900	0.19	−3.15	**2.43**	−2.67	Similar to WRKY 1 (Fragment).	GO:0003700, GO:0006355, GO:0043565
OS05G0322900	1.25	−1.98	**3.04**	−0.17	Similar to WRKY transcription factor 45.	GO:0003700, GO:0009862, GO:0009864, GO:0010200, GO:0043565, GO:0045892, GO:0050832, GO:1900056
OS05G0571200	3.60	−0.37	5.70	**2.53**	Similar to WRKY transcription factor 19.	GO:0003700, GO:0006355, GO:0043565
OS11G0117600	3.59	−0.18	5.29	**3.60**	Similar to WRKY transcription factor 50 (Fragment).	GO:0003700, GO:0006355, GO:0043565
OS03G0437200	1.00	−1.14	**5.03**	0.67	C2H2-type zinc finger protein, Abscisic acid-induced antioxidant defence, Water stress and oxidative stress tolerance	GO:0003700, GO:0005634, GO:0006355, GO:0043565, GO:0044212, GO:0046872
OS10G0377300	5.42	0.66	3.21	**−4.34**	Similar to Homeobox-leucine zipper protein HOX8.	GO:0003700, GO:0005634, GO:0006355, GO:0043565
OS12G0555200	3.14	1.10	**6.30**	**3.50**	Similar to Probenazole-inducible protein PBZ1.	GO:0004864, GO:0004872, GO:0005634, GO:0005737, GO:0006952, GO:0009607, GO:0009738, GO:0010427, GO:0043086, GO:0080163
OS12G0555500	2.03	0.42	**4.62**	**2.85**	Pathogen resistance protein PBZ1,C 17 kDa RNase, Disease resistance	GO:0004864, GO:0004872, GO:0005634, GO:0005737, GO:0006952, GO:0009607, GO:0009738, GO:0010427, GO:0043086, GO:0080163
OS12G0555000	3.75	0.52	6.81	**4.27**	Similar to Probenazole-inducible protein PBZ1.	GO:0004864, GO:0004872, GO:0005634, GO:0005737, GO:0009738, GO:0010427, GO:0043086, GO:0080163
OS12G0555300	3.35	0.35	5.23	**2.70**	Similar to Pathogenesis-related protein PR-10a.	GO:0006952, GO:0009607
OS02G0282000	3.52	1.30	4.64	**2.65**	NB-ARC domain containing protein.	GO:0043531
OS01G0134700	2.02	−0.96	3.78	0.29	Calmodulin binding protein-like family protein.	GO:0002229, GO:0003700, GO:0005516, GO:0005634, GO:0006355, GO:0010112, GO:0010224, GO:0043565, GO:0071219, GO:0080142, GO:1900426
OS01G0508100	2.48	−1.35	4.13	0.89	Ferritin/ribonucleotide reductase-like family protein.	GO:0010112
OS02G0121700	3.68	−0.23	4.63	**2.82**	Terpenoid synthase domain containing protein.	GO:0000287, GO:0009507, GO:0010333, GO:0016114, GO:0034007, GO:0042742, GO:0043693
OS02G0570400	3.55^**c**^	−0.11	5.06	**2.08**	Similar to Ent-kaurene synthase 1A.	GO:0000287, GO:0006952, GO:0008152, GO:0010333, GO:0034277
OS03G0103300	3.42	−1.76	4.33	**1.68**	Hybrid proline- or glycine-rich protein, Control of low-temperature germinability, Pre-harvest sprouting resistance	GO:0005783, GO:0009506, GO:0009707, GO:0050832
OS04G0226600	1.63	−1.76	**3.56**	−0.63	Similar to OSIGBa0135L04.3 protein.	GO:0004674, GO:0005524, GO:0005886, GO:0006468, GO:0006952, GO:0009506
OS05G0368000	4.03	−1.95	5.31	0.50	Non-protein coding transcript.	GO:0010112
OS07G0537900	2.97	−0.27	3.49	0.77	Similar to SRK3 gene.	GO:0004674, GO:0005524, GO:0005886, GO:0006468, GO:0006952, GO:0009506, GO:0016021
OS06G0210400	1.63	−2.81	1.79	−1.61	Concanavalin A-like lectin/glucanase, subgroup domain containing protein.	GO:0004674, GO:0005524, GO:0006468, GO:0016021, GO:0030246
OS04G0109100	5.74	−1.52	8.78	**2.92**	Concanavalin A-like lectin/glucanase domain containing protein.	GO:0004674, GO:0005524, GO:0006468, GO:0030246
OS04G0370900	3.91	−1.01	5.03	0.77	Similar to H0607F01.5 protein.	GO:0004672, GO:0005509, GO:0005515, GO:0005524, GO:0006468, GO:0016021, GO:0030247
OS04G0371000	4.22	−1.80	4.98	0.05	Four-helical cytokine family protein.	GO:0030247
OS08G0514000	4.02	0.95	**6.61**	1.49	Protein kinase, catalytic domain containing protein.	GO:0004672, GO:0005524, GO:0006468, GO:0016021, GO:0030246
OS03G0802500	1.90	−1.45	3.13	0.42	ATPase, AAA-type, C core domain containing protein.	GO:0005524, GO:0016021

Notes: ^a^ represents the samples collected at the 22% of soil moisture content; ^b^ represents the samples collected at the 20% of soil moisture content; ^c^ the value represents the log2 transformation of the fold change of the expression

In total, 18 and 13 DEHGs were identified in the modules brown and royalblue, respectively (Fig. 7b, c; Table 4; Table S4). GO analysis revealed that the DEHGs in the module brown play different roles in various biological processes, including response to stress (GO:0006950), response to endogenous stimulus (GO:0009719), protein phosphorylation (GO:0006468), protein metabolic process (GO:0019538), lipid metabolic process (GO:0006629), carbohydrate metabolic process (GO:0005975), protein folding (GO:0005515), regulation of transcription (GO:0006355), and signal transduction (GO:0007165) (Fig. 7b; Table S5–6). Nearly all of these genes were induced at point 22 and were repressed at point 20 in response to drought in both genotypes. Although they have a similar expression pattern, a different extent of downregulation was observed in several hub genes; for example, OS11G0569300, OS01G0971800, OS04G0675400, and OS09G0243200 showed a lower extent of downregulation at point 20 of PY6, while OS07G0201500 and OS11G0209600 showed a lower extent of downregulation at point 20 of PR403 (Fig. 7b; Table S4). In the module royalblue, only one GO term was significantly overrepresented due to the relatively low number of DEHGs. The striking feature of the expression pattern of the hub genes was that their transcripts were dramatically induced by drought at point 22 of PY6, and their levels were also slightly reduced at the next sampling point. Interestingly, Os04g0494100 and Os11g0592200 were annotated as genes encoding PR3 and PR4 family proteins, which have plant chitinase activity. Os03g0663600 and Os12g0630200 are two genes encoding pathogenesis-related thaumatin-like proteins, which are members of the PR5 family, suggesting that these genes played roles in the cross-talk between drought and biotic stresses in this study (Fig. 7c; Table 4; Table S5–7).

**Table 4:** Differentially expressed hub genes identified in the module royalblue

Gene_id	PR403_22^**a**^	PR403_20^**b**^	PY6_22	PY6_20	Description	GO enrichment
Os01g0298400	1.91^**c**^	−0.31	3.15	0.57	Myb transcription factor domain containing protein.	GO:0003677
Os01g0392600	−0.81	−2.14	**2.14**	−2.02	Conserved hypothetical protein.	
Os03g0663600	1.87	0.68	**4.31**	**2.03**	Similar to Pathogenesis-related thaumatin-like protein.	
Os12g0630200	0.44	−2.47	**2.76**	−0.64	Thaumatin%2C pathogenesis-related family protein.	
Os11g0592200	0.33	−2.22	**2.14**	−1.30	Similar to Chitin-binding allergen Bra r 2 (Fragments).	GO:0042742, GO:0050832
Os04g0494100	1.63	0.65	**4.83**	1.19	Similar to Chitinase.	GO:0000272, GO:0004568, GO:0005618, GO:0006032, GO:0006952, GO:0008061, GO:0009611, GO:0009617, GO:0010262, GO:0016998
Os06g0487620	1.97	1.70	2.31	0.98	Conserved hypothetical protein.	
Os06g0493100	5.99	0.26	5.93	1.68	Conserved hypothetical protein.	GO:0016021
Os06g0586300	−0.01	1.13	**5.08**	**3.48**	Hypothetical protein.	
Os06g0588450	−1.00	−1.82	**4.64**	**2.74**	Hypothetical conserved gene.	
Os06g0591200	4.28	−2.17	**8.51**	**4.08**	Conserved hypothetical protein.	

Notes: ^a^ represents the samples collected at the 22% of soil moisture content; ^b^ represents the samples collected at the 20% of soil moisture content; ^c^ the value represents the log2 transformation of the fold change of the expression

## Discussion

In this study, CSSL PY6 was used to characterize a QTL locus, *dss-1*, for its drought-sensitive phenotype and to investigate the impact of *dss-1* on the reprogramming of transcriptional profiles of PY6 in response to drought stress via RNA-seq and WGCNA.

### Drought-induced overaccumulation of H_2_O_2_ may lead to the sensitive phenotype of *dss-1*

Reactive oxygen species (ROS) (e.g., O^2.-^, H_2_O_2_, OH˙, ^1^O_2_) are unavoidable toxic byproducts of plants generated in response to abiotic/biotic stresses [66]. The overaccumulation of ROS in cells causes severe oxidative damage to membranes (lipid peroxidation), proteins, RNA, and DNA molecules [67]. Higher plant has thus evolved dedicated scavenging pathways to protect themselves from ROS toxicity, including pathways involving detoxifying enzymes such as CAT, SOD, POD, and APX, as well as the antioxidant ascorbate-glutathione (GSH) cycle [19, 68]. In this study, significant overaccumulation of H_2_O_2_ was detected in PY6 compared with PR403 during drought treatment (Fig. 2a, b). Regarding the drought-sensitive phenotype of PY6, which was afforded by the introgression of *dss-1* into the background of PR403, our results suggested that important relationships were evident among *dss-1*, H_2_O_2_ accumulation, and the sensitive phenotype. That is, we speculated that the introgression of *dss-1* might directly or indirectly lead to the overaccumulation of H_2_O_2_ in rice cells. The overaccumulation of H_2_O_2_ causes oxidative damage to the cell membrane (lipid peroxidation), which was reflected by the measurements of MDA and others, subsequently leading to the death of rice plants under severe drought stress conditions.

### Photosynthesis regulation, a common regulatory process for rice in response to drought stress

Chloroplasts are important organelles for the fixation of light energy necessary for the biological activity of higher plant and all other life forms in our biosphere [69]. Beyond their normal role in photosynthesis, increasing evidence suggests that chloroplasts are targeted organelles that are involved in environmental stress responses by positively or passively tuning photosynthetic activity to adapt to environmental changes [24]. According to the GO enrichment analysis, the drought-induced DEGs identified either in PR403 or PY6 were mainly enriched in photosynthesis-related GO terms (Fig. 4; Figs. S4; S5). Additionally, the analysis of differentially expressed hub genes that clustered in the modules black and blue showed that a similar set of GO terms that are related to the photosynthesis process in chloroplasts were overrepresented (Fig. S9A, B). In terms of the altered expression during drought treatment, in line with the results of previous studies [59], the majority of hub genes clustering in the modules black and blue consistently declined to a similar extent in both PY6 and PR403 (Fig. S8A, B), implying that the photosynthetic activity of both genotypes was dramatically inhibited in response to drought stress. The inhibition of photosynthesis reduces the utilization of absorbed light energy; excess light energy results in the generation of toxic ROS [17]. Therefore, photosynthetic activity regulation could be a common regulatory process that is shared among diverse varieties for the adaptation of rice in response to environmental stresses. The inhibition of photosynthesis in both genotypes of this study might be correlated to the accumulation of H_2_O_2_ in rice cells. The differential accumulation of H_2_O_2_ in the leaves of PY6 and PR403 results from the effects of the introgression of *dss-1* into PR403 on the different expression changes of the hub genes identified in the related modules. Furthermore, previous studies considered that the improvement of photosynthetic capability was important for drought resistance of plants [42]. However, in this study, no significant expression difference was observed in the photosynthesis-related DEGs identified in either PR403 or PY6, suggesting that the *dss-1*-induced drought-sensitive phenotype was not caused by the severe inhibition of photosynthesis but, rather, by overaccumulation of H_2_O_2_.

### ROS-correlated hub genes that were differentially regulated in response to drought stress

Transcription factors are key players in the regulatory networks of plants in response to unfavorable stresses. The MYB family is one of the largest transcription factor families in plants; its members have a conserved MYB-binding domain, and the MYB family TFs have been shown to be induced by H_2_O_2_ in soybean and play essential roles in response to abiotic stress [70, 71]. In this study, five DEGs annotated as transcription factors, including 3 MYBs, 1 NAD, and 1 SHR, were highly clustered in the module grey60, which was statistically correlated with the accumulation of H_2_O_2_ during drought stress. Among the genes, Os01G0702700, Os05G0543600, and Os04G0508500 are MYB family transcription factors and showed a higher magnitude of upregulation at point 22 of PR403. Considering the relatively low accumulation of H_2_O_2_ and MDA in PR403, MYB family TFs may be induced by drought stress or other signaling messengers and may trigger the activation of downstream components of drought-responsive signaling pathways. This action may also include the upregulation of stress-responsive genes involved in increasing the content of osmo-protectants and the activities of POD, CAT, and SOD, as well as maintaining a sublethal content of H_2_O_2_ in plant cells during drought stress, suggesting that the MYB TFs identified here were positively correlated with drought resistance (Figs. 2 and 6c; Table 2) [72]. Moreover, WRKY TFs, a family of transcription factors involved in plant defense responses [73], were found to be strongly correlated with MDA and 8 differentially expressed WRKY TFs identified in the module red showed a higher degree of induction at point 22 and a lower level at point 20 in PY6 compared with PR403 (Table 3).

PR proteins are another group of proteins that is involved in plant defense responses [38]. In this study, four PR10 family encoding genes identified in the module red and three other families of PR proteins (PR3, PR4, and PR5) identified in the module royalblue exhibited similar altered expression patterns in response to drought stress. Among the identified PR-encoding genes, OS12G0555000 (*RSOsPR10*), a gene encoding the rice root-specific pathogenesis-related protein PBZ1, was previously reported to be induced by drought and salinity stresses, as well as jasmonic acid; however, it was strongly inhibited by salicylic acid [38, 63]. Besides, the expression of *RSOsPR10* was postulated to be repressed by SA-induced *OSTGA*s or *OsWRKY*s [38]. Our results suggested that cross-talk occurs between abiotic and biotic stress-responsive pathways during drought stress. Recent reports have revealed that some common components are shared by the signaling pathways involved in abiotic and biotic stress responses: the phytohormones abscisic acid, salicylic acid, and jasmonic acid; *cis*-acting regulatory elements; and components of protein kinase cascades [2]. Plants can rapidly respond to environmental changes by the synergistic or antagonistic regulation of cross-talk among pathways via their convergent nodes [2]. *OsWRKY76* (OS09G0417600), a hub gene of the module red, encodes a group IIa WRKY TF in rice. It has been shown that overexpression of *OsWRKY76* in rice plants suppresses a specific set of PR genes and increases the expression of abiotic stress-associated genes, which resulted in increased susceptibility to *Magnaporthe oryzae* (*M. oryzae*) and improved tolerance to chilling stress [34]. In addition, *OsWRKY13* was suggested to regulate the antagonistic cross-talk between drought and disease resistance pathways via the repression of *SNAC1* and *WRKY45–1* [33]. Thus, the WRKY TF- and PR family protein-encoded genes identified here may act as convergent nodes and may be responsible for the cross-talk between abiotic and biotic stresses.

**Fig. 8:**
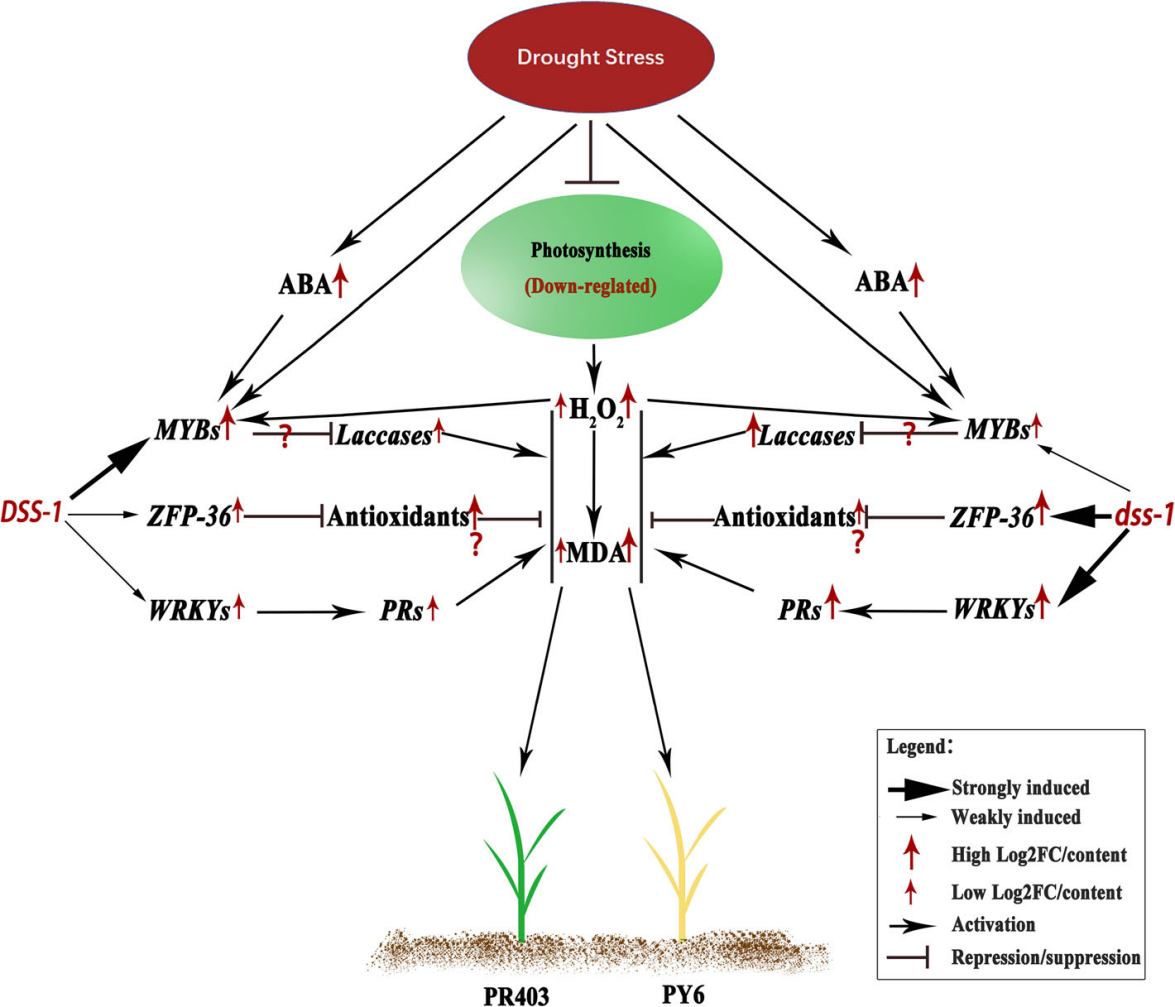
Possible role of *dss-1* in reprogramming the transcriptional profiles of rice in response to drought stress. When rice is exposed to severe drought stress, the inhibition of photosynthesis leads to the overaccumulation of toxic ROS, including H_2_O_2_. ABA and H_2_O_2_ signals are integrated and perceived and trigger the activation of responsive signaling pathways. First, the transcription factors, MYBs, WRKYs, ZFP36, NACs, etc., was differentially reprogrammed. The downstream responsive genes, including a number of PR family proteins and laccases, are then regulated to respond to drought stress, and ameliorating the drought-induced damage of rice. The introgressed *dss-1* may exert its effect on the hub genes and differentially alter their expression, therefore leading to the drought-resistant and -sensitive variation of rice

In addition, strikingly, Os01G0842500 and Os03G0273200, both of which were annotated as putative laccase-encoding genes, consistently exhibited elevated expression in both genotypes in response to drought. However, PY6 had a relatively higher upregulated expression at point 20, implying that the overaccumulation of the transcripts of the genes for laccase may be correlated with the drought-sensitive phenotype of PY6. It has been demonstrated that a rice laccase-encoding gene, *OsChi1*, has been reported to play an important role in the ROS signaling pathway [74], suggesting that, in the present study, MYB TF-regulated laccases with differential expression are involved in the improvement of drought stress tolerance (Fig. 8). However, paradoxically, the overexpression of another laccase-encoding gene, *OsChi1* (Os01g0827300), in *Arabidopsis* resulted in increased tolerance to drought and salinity stresses [74]. Thus, further study needs to be performed to explore the function of the laccases identified in the study and determine the discrepancy. Moreover, *ZFP36* (OS03G0437200), a gene encoding a C_2_H_2_ zinc finger protein (Brs-d1), is a key factor for regulating rice cell redox status and is involved in ABA-induced upregulation of the expression and activity of SOD and APX. Overexpression of *ZFP36* enhanced tolerance to drought and oxidative stresses but reduced resistance to rice blast [64, 65]. However, dramatically increased expression of *ZFP36* was exhibited in the drought-sensitive genotype PY6, suggesting that this gene may play a synergistic role in the regulatory network with WRKY TFs and PRs in rice plants in response to drought stress.

### Impact of *dss-1* on the reprogramming of hub genes in response to drought stress

Taken together, a working model was proposed here to depict the possible *dss-1*-mediated mechanism of rice plants in response to drought stress at the transcriptional level (Fig. 8). When rice is exposed to severe drought stress, stress-induced dehydration of rice plants and stomatal closure of leaf guard cells result in the inhibition of photosynthesis activity and the enhancement of ABA biosynthesis. The inhibition of photosynthesis reduces the utilization of absorbed light energy and results in the generation of toxic ROS, including H_2_O_2_. Overaccumulation of ROS can cause oxidative stress damage to plant cells, leading to the drought-sensitive phenotype of rice. Drought stress signals, ABA accumulation, and H_2_O_2_ signals are integrated and perceived by rice cells and trigger the activation of responsive signaling pathways. First, the expression of a series of TFs, including MYBs, WRKYs, ZFP36, NACs, etc., was differentially reprogrammed, and these TFs induced cross-talk between drought and biotic stress responses. Induction of the TFs then regulates the expression of downstream responsive genes, including a number of PR family proteins and laccases, to mobilize multiple aspects of in vivo resources to respond to drought stress, and eventually reducing the content of ROS in plant cells and ameliorating the drought-induced sensitive phenotype of rice. The introgressed *dss-1* may exert its effect on the hub genes that were identified in our study to differentially alter their expression, therefore leading to the differential accumulation of ROS between drought-resistant and drought-sensitive varieties. The differential activation of ROS-related responsive pathways could cause the differences between the drought resistance/sensitive phenotypes of rice varieties.

In this study, we used a drought sensitive plant material to fine map QTL *dss-1* and investigate its effect on the transcriptomic profiling of rice. In subsequent, we will identify a drought sensitive QTL allele from Lambayeque1 by positional cloning; the drought tolerant allele of *dss-1* from PR403 can also be isolated as well. They will then be studied for the elucidation of drought tolerant mechanisms and drought tolerant allele *DSS-1* will be applied for rice breeding.

## Conclusion

Taken together, our results suggest that chloroplasts are the targeted organelles for PR403 and PY6 in response to drought stress regulation. Functional annotation analysis of the hub genes in the modules that were strongly correlated with H_2_O_2_ and MDA accumulation during drought treatment provided us with a global picture of the impact of *dss-1* on the reprogramming of the regulatory mechanism(s) at the transcriptional level. Specifically, the differential expression patterns of *MYB*s, *WRKY*s, PR protein encoding genes, *ZFP36*, etc., in both genotypes implied their roles in the variation in ROS and MDA accumulation, thereby resulting in the sensitive phenotype of rice in response to drought stress.

## Methods

### Plant materials, growth, and treatment conditions

PY6 was screened in a set of CSSLs that were constructed by Jijing Luo, Jianbin Liu, and Baiyang Yu by introgression of the genomic fragments of donor parent Lambayeque 1 into recurrent parent PR403. The parental lines, PR403 (Accession number: AAV003680) and Lambayeque 1 (Accession number: AAV003699) are deposited in Genetic resource bank of Shanghai Agricultural Biological Gene Center (http://seed.sagc.org.cn/front/custom/enterViewResourceList.action?gerTypeId=4028940b44ba2d910144ba3062d00002&gerTypeCode=AA&gerTypeName=%E6%B0%B4%E7%A8%BB%E7%B1%BB&isFeature=0). All the plant materials were grown in compliance with the legislation of China in the experimental paddy fields of Guangxi University according to previously described methods with minor modifications [16]. In brief, rice plants were grown in equal amounts of paddy soil. Additionally, an equal amount of water was routinely applied. The drought treatment was performed according to previously described methods [25]. In the 17-day of drought treatment, soil moisture was monitored regularly to determine the stages of drought stress using an EM-WSYP soil moisture detector (Hengmei Company, China). The penultimate leaves of 3-week-old soil-cultured rice seedlings (three biological replicates) grown in the greenhouse (35 °C day/30 °C night) were collected at four soil moisture points, 40 (40% soil moisture, representing well-watered conditions), 22 (22% soil moisture, drought treatment for 96 h after 40% soil moisture conditions), 20 (20% soil moisture, drought treatment for 120 h), and 15 (15% soil moisture, drought treatment for 168 h) (Fig. S1), and flash frozen in liquid nitrogen for RNA-seq and physiological index measurements. The samples harvested at point 15 were for physiological measurement analyses only. Drought stress treatment for hydroponically cultivated seedlings was performed in rice nutrient solution supplemented with 20% (w/v) PEG 6000 in 96-well plates. The survival rate was determined after 5 days of treatment and rewatering for 3 days.

### RNA-seq library construction and transcriptomic data processing

A transcriptome library was generated by a NEBNext® Ultra™ RNA Library Prep Kit for Illumina® (NEB, USA), following the manufacturer’s instructions. After digestion, purification, and PCR amplification, the cDNA fragments (250 ~ 300 bp) were purified by AMPure XP beads (Beckman, USA). cDNA sequencing was performed on an Illumina 2500 platform, and paired-end reads were generated. After removing low-quality reads from the data, the read depth of 6 Gb-clean reads that is sufficient for optimal transcriptome coverage universally were obtained and mapped to the reference genome (the Rice Annotation Project Database, https://rapdb.dna.affrc.go.jp/index.html). DEGs between each treatment point and untreated controls were detected using DESeq, with |log2FC| ≥1 (FC, the abbreviation for fold change) and *padj* ≤ 0.05 used as thresholds to identify drought-induced DEGs. Additionally, the development-dependent DEGs were detected in the same sampling points of well-watered samples with the same criteria. Because the development-dependent DEGs could be caused by the development of rice plants, they were excluded from the final dataset.

GO enrichment analysis was performed via PlantRegMap (http://plantregmap.cbi.pku.edu.cn/). The overrepresented GO terms were visualized using ggplot2 and the GOplot R package [75]. A heatmap was generated by MEV v4.9 [76].

### Coexpression network analysis

The WGCNA R package was used to construct a coexpression network and analyze the correlation of modules and physiological data according to a previously described method [47]. Raw transcriptomic datasets of all samples were filtered to remove all genes with an FPKM (Fragments Per Kilobase Million) < 1, even for a single replicate of any sampling point of the samples. Clean data were used to generate a coexpression network. First, the soft threshold power was estimated by the pickSoftThreshold function, which provides the appropriate soft-thresholding power for network construction. Second, a correlation matrix was constructed based on the obtained soft threshold power, and afterward, a topological overlap matrix (TOM) was calculated from the transformed correlation matrix. Finally, the genes were grouped based on the topological overlap dissimilarity (1-TOM) by average hierarchical clustering using the hclust function. Gene modules were then identified using a dynamic tree cutoff algorithm (minimum cluster size of 30, merging threshold function of 0.25). Module membership (MM) was calculated based on Pearson correlations between the expression level and the module eigengenes to identify hub genes within the modules. A relatively high MM indicates that those genes have a relatively high connectivity within the module.

To correlate the physiological data with the network, module eigengenes were correlated with each physiological data point. Gene significance (GS) was used to correlate the physiological data with the expression data of individual genes.

### Analysis of the movement of the stomatal aperture

Four-week-old seedlings were selected for scanning electron microscopy (SEM) analysis. The middle part of penultimate leaves was cut into 0.5-cm pieces, which were subsequently flash frozen in liquid nitrogen to maintain stomatal morphology. An FEI Quattro S scanning electron microscope (Thermo Fisher Scientific, USA) with a freezing stage was used to examine the leaf stomatal apertures. The opening status and density of stomata were analyzed with ImageJ software. Four random fields were selected for counting the number of stomata in each replicate.

### Quantitative real-time reverse transcription PCR analysis

Total RNA was isolated from leaf samples (three biological replicates per sample). cDNA synthesis was performed by reverse transcription (RT) with a Thermo Scientific Revert Aid First Strand cDNA Synthesis Kit (Cat# K1622) according to the manufacturer’s protocol. The sequences of genes were downloaded from RAP-DB (https://rapdb.dna.affrc.go.jp/index.html). The sequences of the primers of the relevant genes were obtained from qPrimerDB (https://biodb.swu.edu.cn/qprimerdb/) (Table S1) [77]. qPCR was performed on a Roche LightCycler 480 Real-Time PCR System in 10 μL reactions with a SYBR green PCR Master Mix Kit (BIO-RAD, USA), following the manufacturer’s protocol. The relative expression of each gene was calculated according to the 2^-ΔΔCT^ method [78]. The *actin* gene (Os11g0163100) was used as an internal reference for analysis.

### Determination of the relative water content of the leaves

Leaf samples were collected at each sampling point and weighed to determine the fresh weight (FW). The samples were then oven-dried to a constant dry weight (DW). The relative water content was subsequently calculated according to the following equation: RWC (%) = [(FW– DW) / FW] × 100%.

### Examination of ABA, ROS and enzyme scavenging activity

Fresh leaf samples (0.15 g) of PR403 and PY6 were harvested and ground in liquid nitrogen into a fine powder, after which they were resuspended in 1.35 mL PBS buffer (10 mM, pH 7.2). The supernatant was used for subsequent assays after centrifugation at 5000 rpm for 5 min. The contents and activity of H_2_O_2_, SOA, ABA, SOD, CAT, POD, and APX were measured using an enzyme-linked immunosorbent assay (ELISA) kit (MSKBIO, China) following the manufacturer’s instructions.

3,3′-Diaminobenzidine (DAB) and nitro blue tetrazolium (NBT) staining were performed to detect the accumulation of H_2_O_2_ and SOA in the leaves following the previously described methods [16].

## Supplementary information

**Additional file 1: Figure. S1.** Diagram showing the sampling points and the corresponding phenotypes of the plant materials.

**Additional file 2: Figure S2.** Determination of APX activity, endogenous ABA level, and stomatal status of PR403 and PY6 under drought treatment. (A) APX activity. (B) CAT activity. (C) NBT staining of leaf samples. (D) Endogenous ABA content. (E) Leaf stomatal opening status during drought treatment. The top panel shows three levels of stomatal aperture: completely open, partially open and completely closed. The bottom panel shows the percentages of three levels of stomatal opening in PY6 and PR403 (*n* = 100 stomata for PR403 and PY6). The different letters at the top of each column in (A), (B), (D), and (E) indicate statistically significant differences based on ANOVA with Tukey’s HSD test (*P* < 0.05). Scale bars = 0.5 cm in (B) and = 5 μm in (D).

**Additional file 3: Figure S3.** Validation of the RNA-seq data by qRT-PCR. The *ACTIN* gene (Os11g0163100) was used as an endogenous reference for qPCR. Os01g0164600 (A), Os01g0289600 (B), Os02g0115700 (C), Os03g0319400 (D), and Os04g0610400 (E) were selected for qPCR. The sequences of the primers used are shown in Table S1.

**Additional file 4: Figure S4.** Cellular component GO terms significantly overrepresented in the DEG GO enrichment analysis. (A) DEG-enriched GO terms at sampling point 22 of PR403. (B) DEG-enriched GO terms at sampling point 22 of PY6. (C) DEG-enriched GO terms at sampling point 20 of PR403. (D) DEG-enriched GO terms at sampling point 20 of PY6. Bubble size is proportional to the number of each GO-term, and the color represents the -log10 (Qvalue).

**Additional file 5: Figure S5.** Molecular function GO terms significantly overrepresented in the DEG GO enrichment analysis. (A) DEG-enriched GO terms at sampling point 22 of PR403. (B) DEG-enriched GO terms at sampling point 22 of PY6. (C) DEG-enriched GO terms at sampling point 20 of PR403. (D) DEG-enriched GO terms at sampling point 20 of PY6. Bubble size is proportional to the number of each GO-term, and the color represents the -log10 (Qvalue).

**Additional file 6: Figure S6.** Soft threshold power estimation in the WGCNA.

**Additional file 7: Figure S7.** Scatter plot of module eigengenes in the modules significantly correlated with H2O2 and MDA. (A) H2O2 accumulation correlated with the modules black, blue, green, and grey60. (B) MDA accumulation correlated with the modules red, brown, and royalblue.

**Additional file 8: Figure S8.** Heatmap showing that the expression patterns of the hub genes in the modules correlated with H2O2 and MDA accumulation. (A) Module Black. (B) Module Blue. (C) Module Grey60. (D) Module Green. (E) Module Red. (F) Module Brown. All the data used in the analysis were subjected to log2 transformation.

**Additional file 9: Figure S9.** GO terms overrepresented in the GO enrichment analysis of hub genes in the modules correlated with H2O2 accumulation. The GO terms of biological processes, cellular components, and molecular functions were overrepresented in the modules (A) black, (B) blue, (C) grey60, and (D) green, respectively. Bubble size is proportional to the number of each GO-term, and the color represents the -log10 (*Qvalue*).

**Additional file 10: Figure S10.** GO terms overrepresented in the GO enrichment analysis of hub genes in the modules correlated with MDA accumulation. The GO terms of biological processes, cellular components, and molecular functions were overrepresented in the modules (A) red, (B) brown, and (C) royalblue, respectively. Bubble size is proportional to the number of each GO-term, and the color represents the -log10 (Qvalue).

**Additional file 11: Table S1.** The primer sequences of genes using for qPCR.

**Additional file 12: Table S2–1.** The differentially expressed genes (DEGs) identified in the comparison of PR403_CK22vsPR403_CK40 (log2FC ≥ 1 and *padj* ≤ 0.05). **Table S2–2.** The differentially expressed genes (DEGs) identified in the comparison of PR403_CK20vsPR403_CK40 (log2FC ≥ 1 and *padj* ≤ 0.05). **Table S2–3.** The differentially expressed genes (DEGs) identified in the comparison of PY6_CK22vsPY6_CK40 (log2FC ≥ 1 and *padj* ≤ 0.05). **Table S2–4.** The differentially expressed genes (DEGs) identified in the comparison of PY6_CK20vsPY6_CK40 (log2FC ≥ 1 and *padj* ≤ 0.05). **Table S2–5.** The development dependent DEGs identified in the comparison of PR403_CK22vsPR403_CK40 (log2FC ≥ 1 and *padj* ≤ 0.05). **Table S2–6** The development dependent DEGs identified in the comparison of PR403_CK20vsPR403_CK40 (log2FC ≥ 1 and *padj* ≤ 0.05). **Table S2–7.** The development dependent DEGs identified in the comparison of PY6_CK22vsPY6_CK40 (log2FC ≥ 1 and *padj* ≤ 0.05). **Table S2–8.** The development dependent DEGs identified in the comparison of PY6_CK20vsPY6_CK40 (log2FC ≥ 1 and *padj* ≤ 0.05). **Table S2–9.** The differentially expressed genes (DEGs) identified in the comparison of PR403_22vsPR403_CK40 (log2FC ≥ 1 and *padj ≤* 0.05). **Table S2–10.** The differentially expressed genes (DEGs) identified in the comparison of PR403_20vsPR403_CK40 (log2FC ≥ 1 and *padj* ≤ 0.05). **Table S2–11.** The differentially expressed genes (DEGs) identified in the comparison of PY6_22vsPY6_CK40 (log2FC ≥ 1 and *padj* ≤ 0.05). **Table S2–12.** The differentially expressed genes (DEGs) identified in the comparison of PY6_20vsPY6_CK40 (log2FC ≥ 1 and *padj* ≤ 0.05).

**Additional file 13: Table S3–1.** Hub genes list of module black. **Table S3–2.** Hub genes list of module blue. **Table S3–3.** Hub genes list of module grey60. **Table S3–4.** Hub genes list of module green. **Table S3–5.** Hub genes list of module red. **Table S3–6.** Hub genes list of module brown. **Table S3–7.** Hub genes list of module royalblue.

**Additional file 14: Table S4.** Differentially expressed hub genes identified in module brown.

**Additional file 15: Table S5–1.** GO enrichment result of module black. **Table S5–2.** GO enrichment result of module blue. **Table S5–3.** GO enrichment result of module grey60. **Table S5–4.** GO enrichment result of module green. **Table S5–5.** GO enrichment result of module red. **Table S5–6.** GO enrichment result of module brown. **Table S5–7.** GO enrichment result of module royalblue. **Table S5–8** GO enrichment result of PR403 22 vs PR403 CK40. **Table S5–9.** GO enrichment result of PR403 20 vs PR403 CK40. **Table S5–10.** GO enrichment result of PY6 22 vs PY6 CK40. **Table S5–11.** GO enrichment result of PY6 20 vs PY6 CK40.

## Data Availability

All data generated or analysed during this study are included in this published article [and its supplementary information files]. The sequencing data is deposited to gene expression omnibus (GEO) database of NCBI (accession number: GSE158928; https://www.ncbi.nlm.nih.gov/geo/query/acc.cgi?acc=GSE158928).

## Abbreviations

CSSL: Chromosome single-substitution segment line
DEGs: Differentially expressed genes
SNPs: Single nucleotide polymorphisms
MDA: Malondialdehyde
WGCNA: Weighted gene coexpression network analysis
ROS: Reactive oxygen species
SOD: Superoxide dismutase
CAT: Catalase
APX: Ascorbate peroxidase
TFs: Transcription factors
PR: Pathogenesis-related
POD: Peroxidase
PUFA: Polyunsaturated fatty acid
PCA: Principal component analysis
BP: Biological process
CC: Cellular component
MF: Molecular function
DEHGs: Differentially expressed hub genes
TOM: Topological overlap matrix
MM: Module membership
GS: Gene significance
SEM: Scanning electron microscopy
FW: Fresh weight
DW: Dry weight
DAB: 3,3′-Diaminobenzidine
NBT: Nitro blue tetrazolium
