# Correction to: Weighted gene coexpression network analysis-based identification of key modules and hub genes associated with drought sensitivity in rice

**DOI:** 10.1186/s12870-020-02730-8

**Published:** 2020-11-10

**Authors:** Baiyang Yu, Jianbin Liu, Di Wu, Ying Liu, Weijian Cen, Shaokui Wang, Rongbai Li, Jijing Luo

**Affiliations:** 1 College of Life Science and Technology (State Key Laboratory for Conservation and Utilization of Subtropical Agro-bioresources), Guangxi University, Nanning, 530004 China; 2 Agriculture College, Guangxi University, Nanning, 530004 China; 3 Agriculture College, South China Agricultural University, Guangzhou, 510642 China

**Correction: BMC Plant Biol (2020) 20:478**

**https://doi.org/10.1186/s12870-020-02705-9**

Correction

In the original publication of this article [1] the author would like to correct the author affiliation sequence and affiliation of Rongbai Li and Jijing Luo. In this correction article the author and the corresponding details are provided.

Baiyang Yu^1†^, Jianbin Liu^1†^, Di Wu^1^, Ying Liu^1^, Weijian Cen^1^, Shaokui Wang^3^, Rongbai Li^1,2*^ and Jijing Luo^1*^

1. College of Life Science and Technology (State Key Laboratory for Conservation and Utilization of Subtropical Agro-bioresources), Guangxi University, Nanning 530004, China

2. Agriculture College, Guangxi University, Nanning 530004, China

3. Agriculture College, South China Agricultural University, Guangzhou 510642, China

The original publication has been corrected.

## References

[1] Yu B (2020). Weighted gene coexpression network analysis-based identification of key modules and hub genes associated with drought sensitivity in rice. BMC Plant Biol.

