# Supplementary figures and images for: Weighted gene coexpression network analysis-based identification of key modules and hub genes associated with drought sensitivity in rice
Source: BMC Plant Biol. 2020 Oct 20;20:478. doi: 10.1186/s12870-020-02705-9 (PMC7576772; doi:10.1186/s12870-020-02705-9)

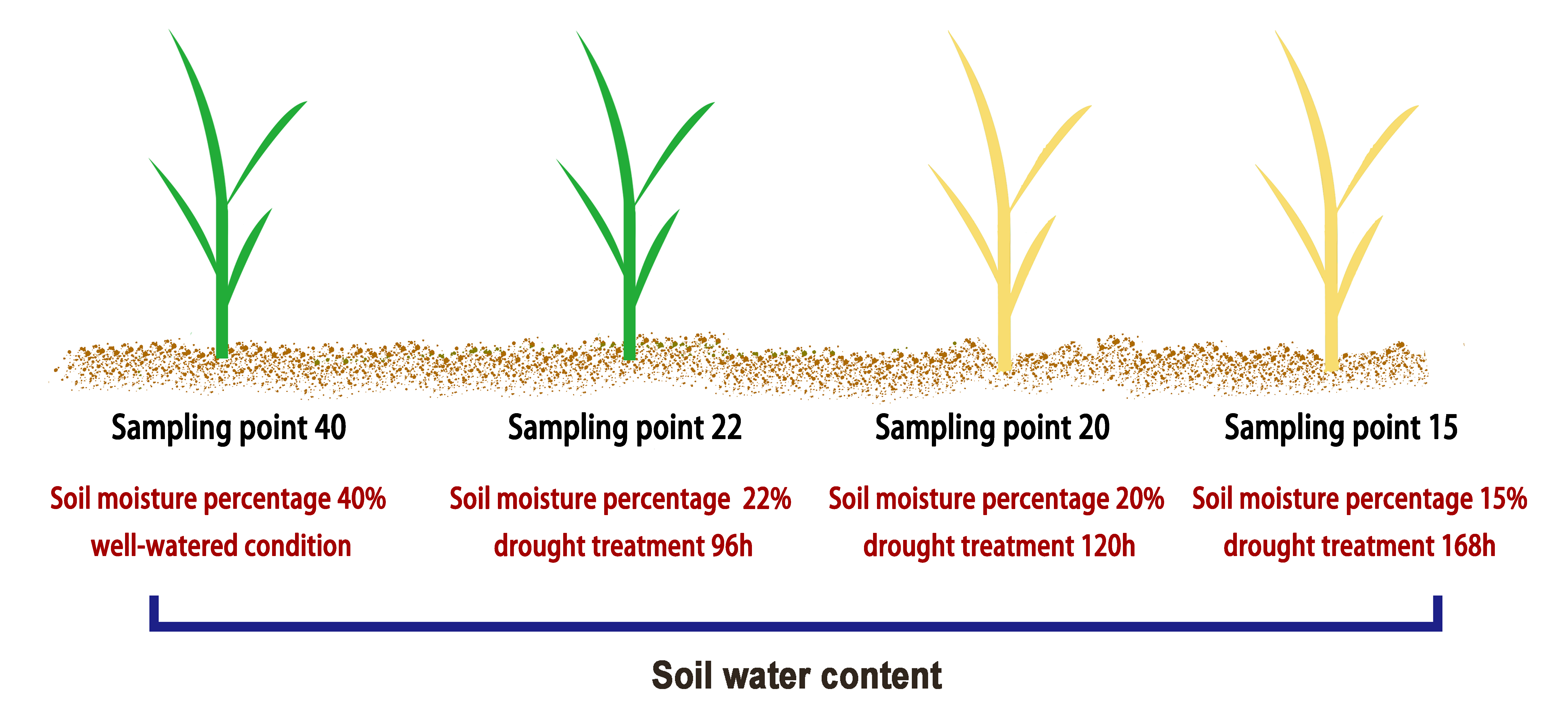

Supplement: Supplementary file 1 — Additional file 1: Figure. S1. Diagram showing the sampling points and the corresponding phenotypes of the plant materials. [file 12870_2020_2705_MOESM1_ESM.tif]

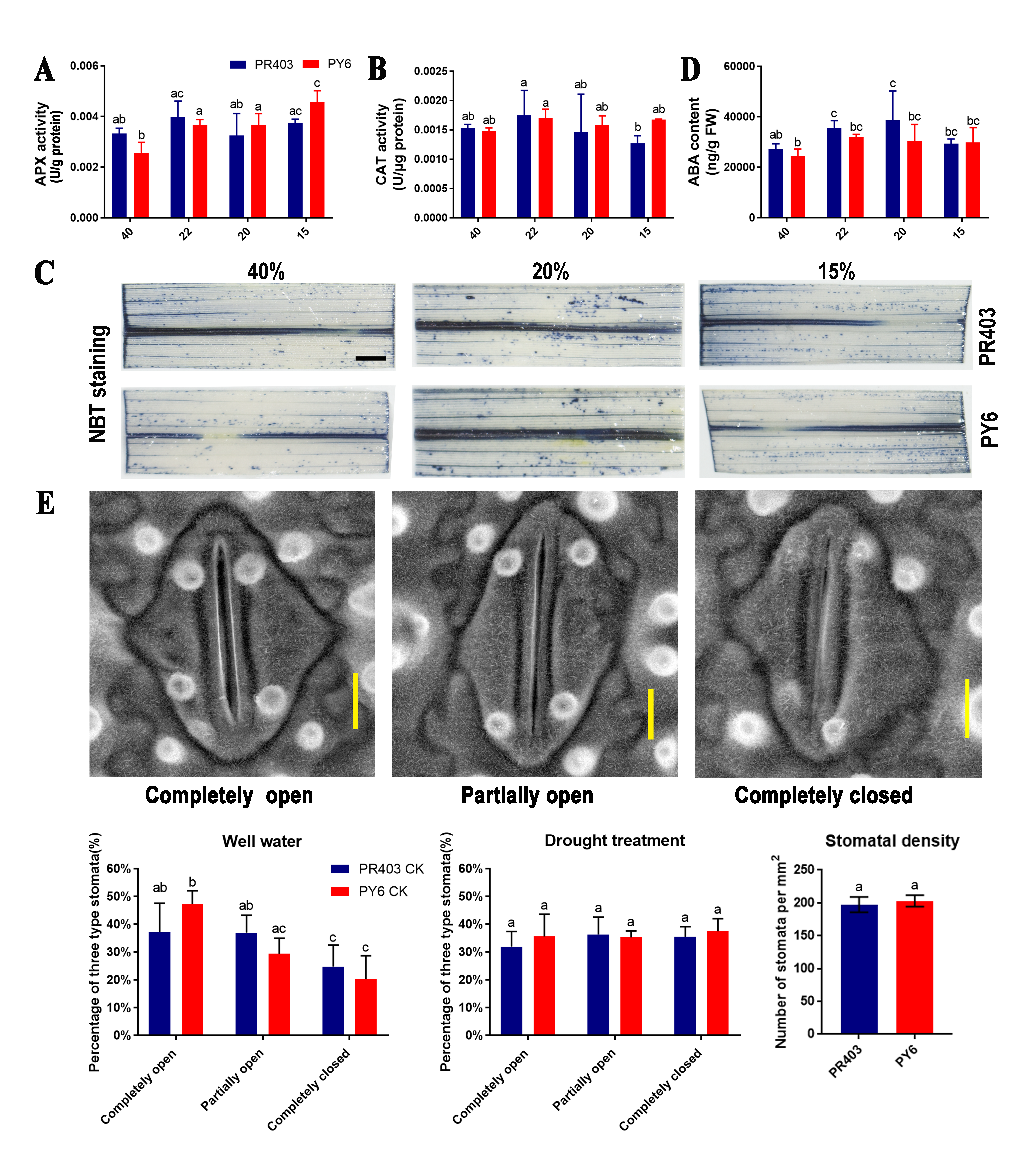

Supplement: Supplementary file 2 — Additional file 2: Figure S2. Determination of APX activity, endogenous ABA level, and stomatal status of PR403 and PY6 under drought treatment. (A) APX activity. (B) CAT activity. (C) NBT staining of leaf samples. (D) Endogenous ABA content. (E) Leaf stomatal opening status during drought treatment. The top panel shows three levels of stomatal aperture: completely open, partially open and completely closed. The bottom panel shows the percentages of three levels of stomatal opening in PY6 and PR403 (n = 100 stomata for PR403 and PY6). The different letters at the top of each column in (A), (B), (D), and (E) indicate statistically significant differences based on ANOVA with Tukey’s HSD test (P < 0.05). Scale bars = 0.5 cm in (B) and = 5 μm in (D). [file 12870_2020_2705_MOESM2_ESM.tif]

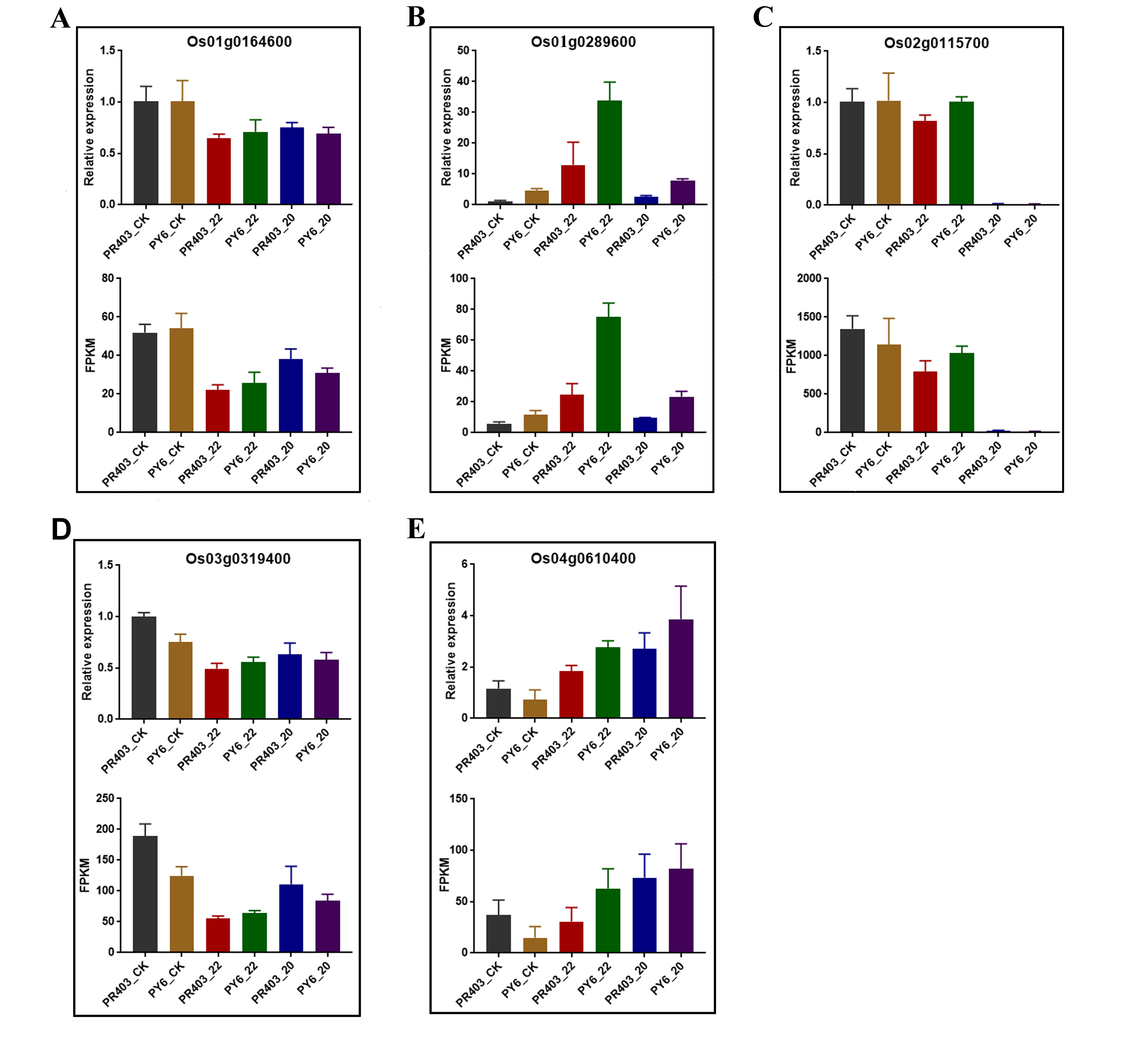

Supplement: Supplementary file 3 — Additional file 3: Figure S3. Validation of the RNA-seq data by qRT-PCR. The ACTIN gene (Os11g0163100) was used as an endogenous reference for qPCR. Os01g0164600 (A), Os01g0289600 (B), Os02g0115700 (C), Os03g0319400 (D), and Os04g0610400 (E) were selected for qPCR. The sequences of the primers used are shown in Table S1. [file 12870_2020_2705_MOESM3_ESM.tif]

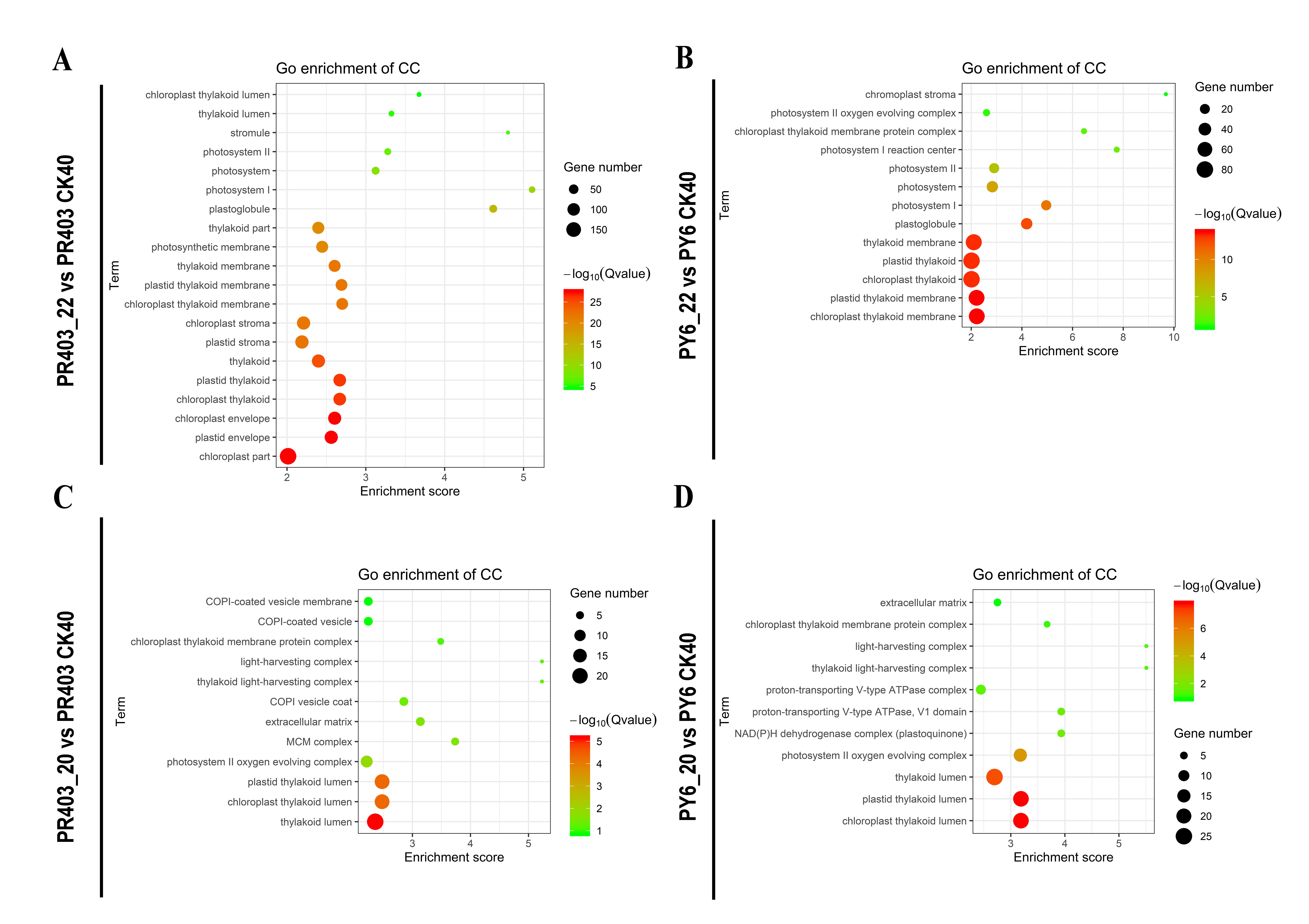

Supplement: Supplementary file 4 — Additional file 4: Figure S4. Cellular component GO terms significantly overrepresented in the DEG GO enrichment analysis. (A) DEG-enriched GO terms at sampling point 22 of PR403. (B) DEG-enriched GO terms at sampling point 22 of PY6. (C) DEG-enriched GO terms at sampling point 20 of PR403. (D) DEG-enriched GO terms at sampling point 20 of PY6. Bubble size is proportional to the number of each GO-term, and the color represents the -log10 (Qvalue). [file 12870_2020_2705_MOESM4_ESM.tif]

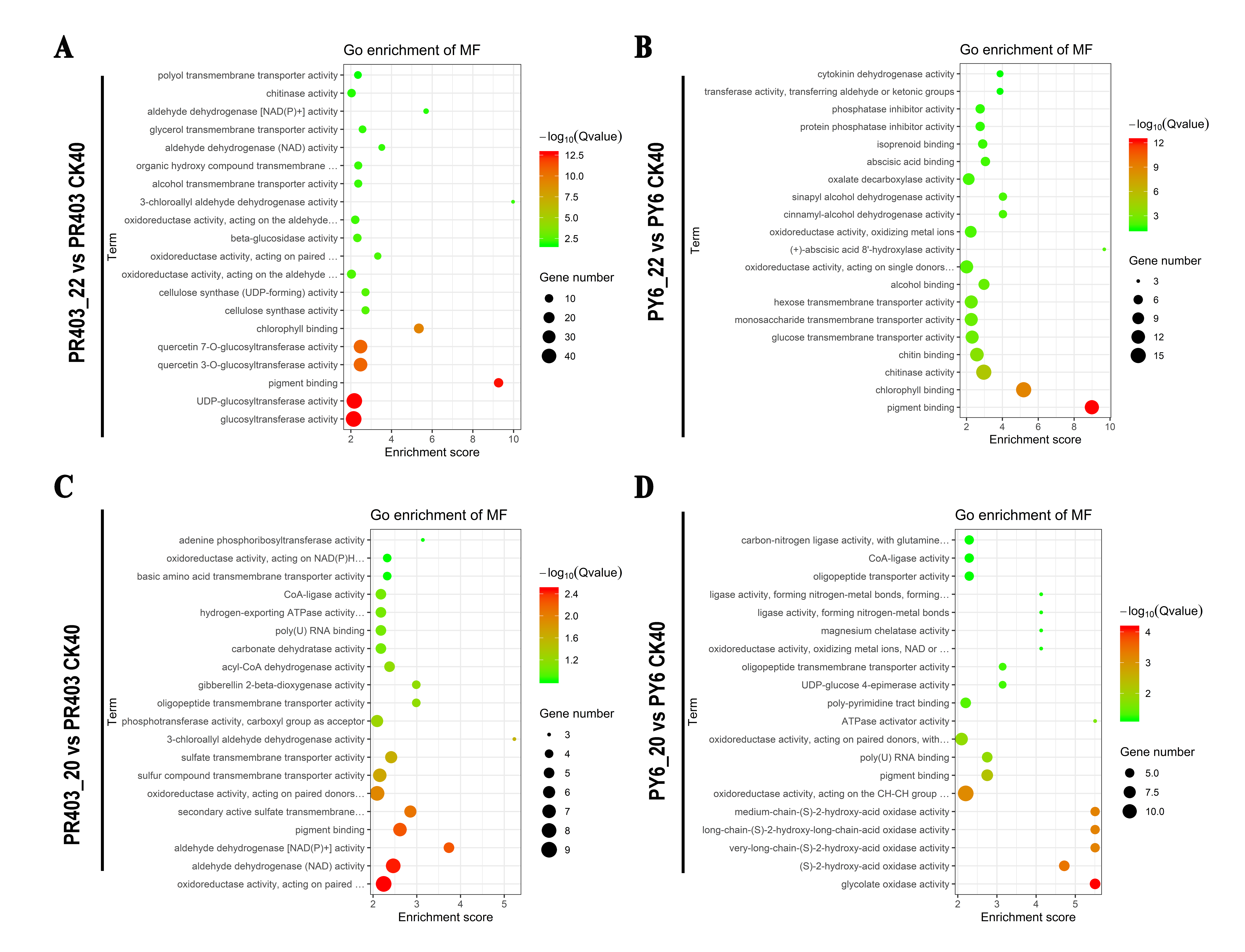

Supplement: Supplementary file 5 — Additional file 5: Figure S5. Molecular function GO terms significantly overrepresented in the DEG GO enrichment analysis. (A) DEG-enriched GO terms at sampling point 22 of PR403. (B) DEG-enriched GO terms at sampling point 22 of PY6. (C) DEG-enriched GO terms at sampling point 20 of PR403. (D) DEG-enriched GO terms at sampling point 20 of PY6. Bubble size is proportional to the number of each GO-term, and the color represents the -log10 (Qvalue). [file 12870_2020_2705_MOESM5_ESM.tif]

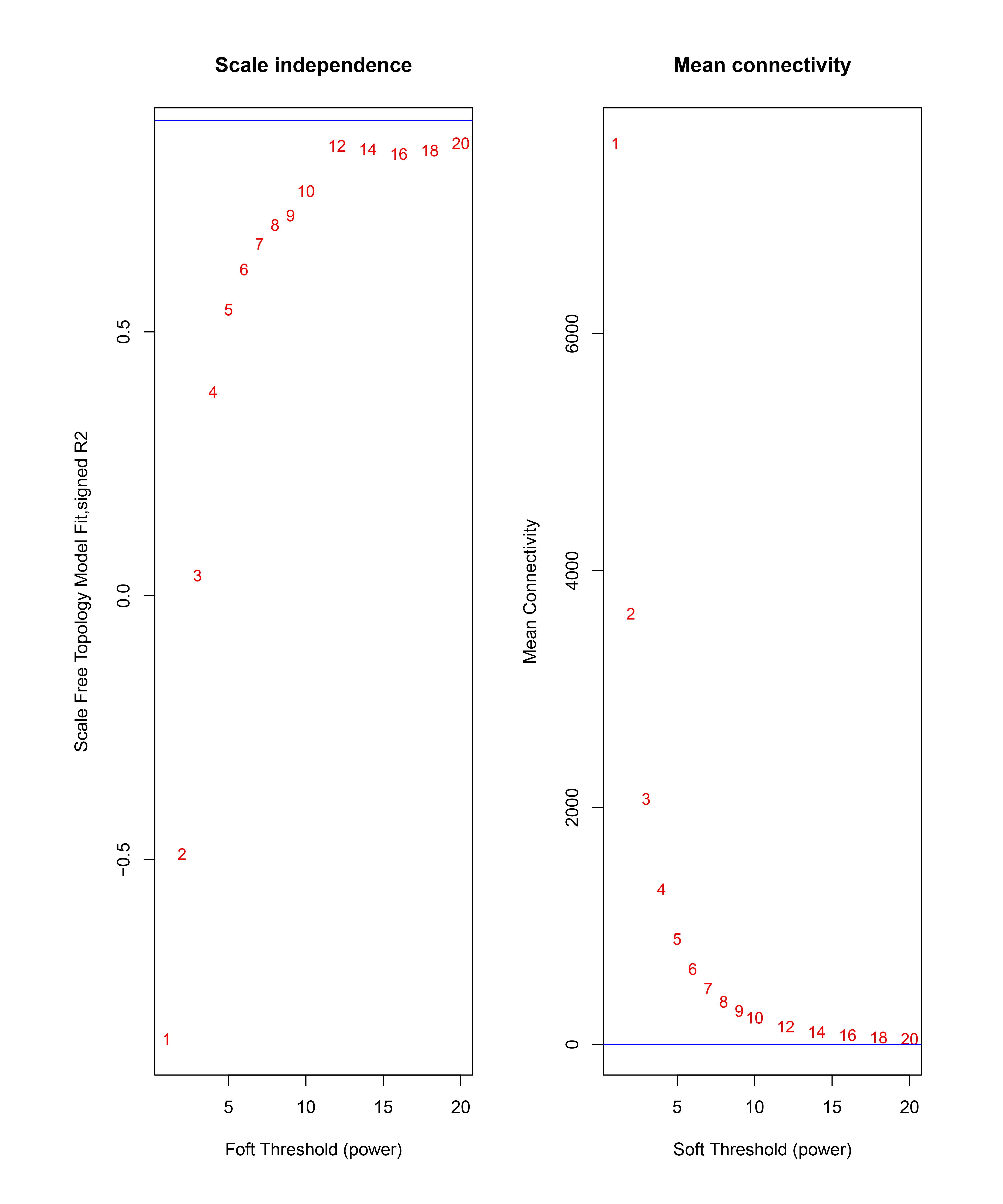

Supplement: Supplementary file 6 — Additional file 6: Figure S6. Soft threshold power estimation in the WGCNA. [file 12870_2020_2705_MOESM6_ESM.tif]

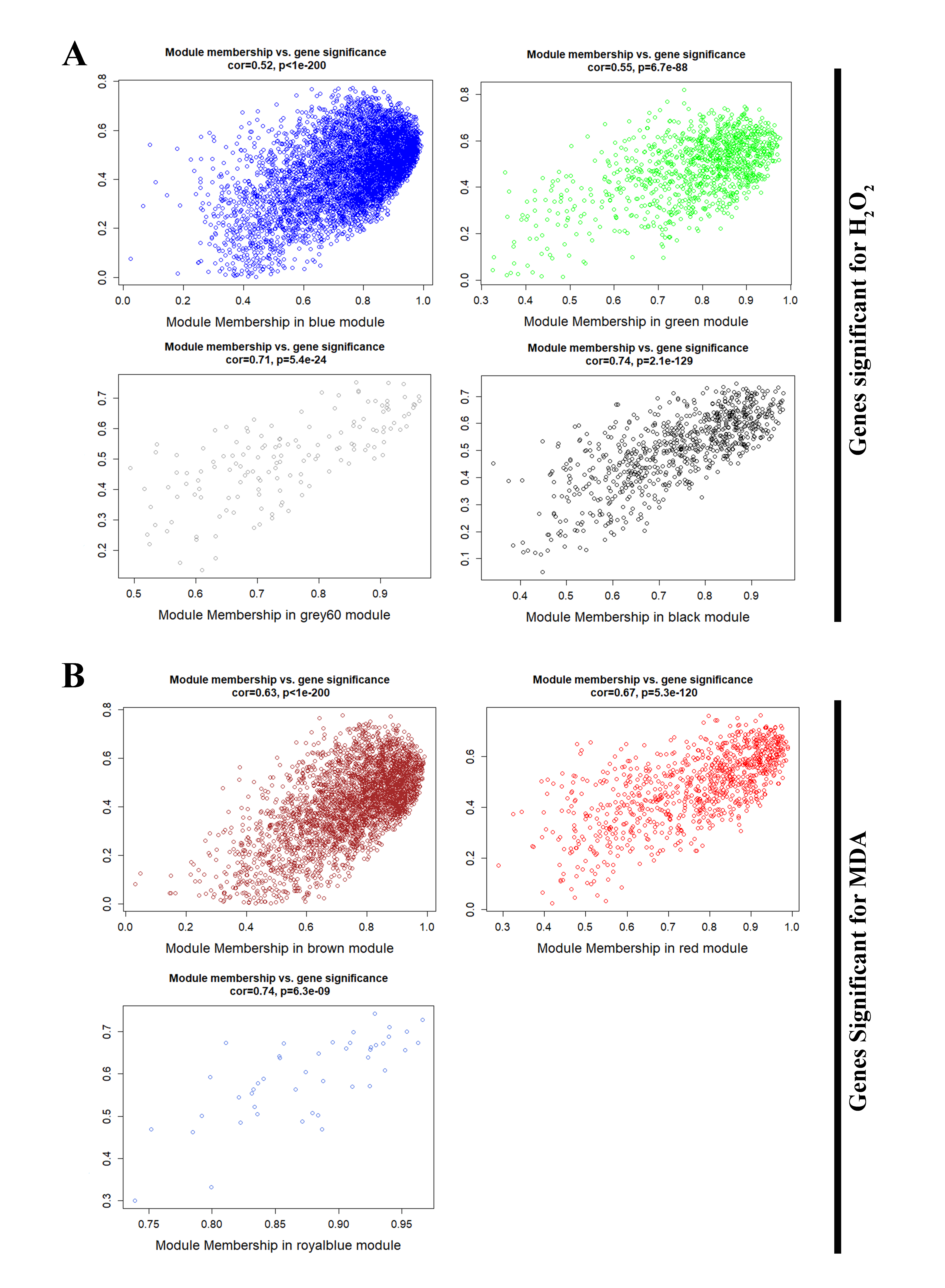

Supplement: Supplementary file 7 — Additional file 7: Figure S7. Scatter plot of module eigengenes in the modules significantly correlated with H2O2 and MDA. (A) H2O2 accumulation correlated with the modules black, blue, green, and grey60. (B) MDA accumulation correlated with the modules red, brown, and royalblue. [file 12870_2020_2705_MOESM7_ESM.tif]

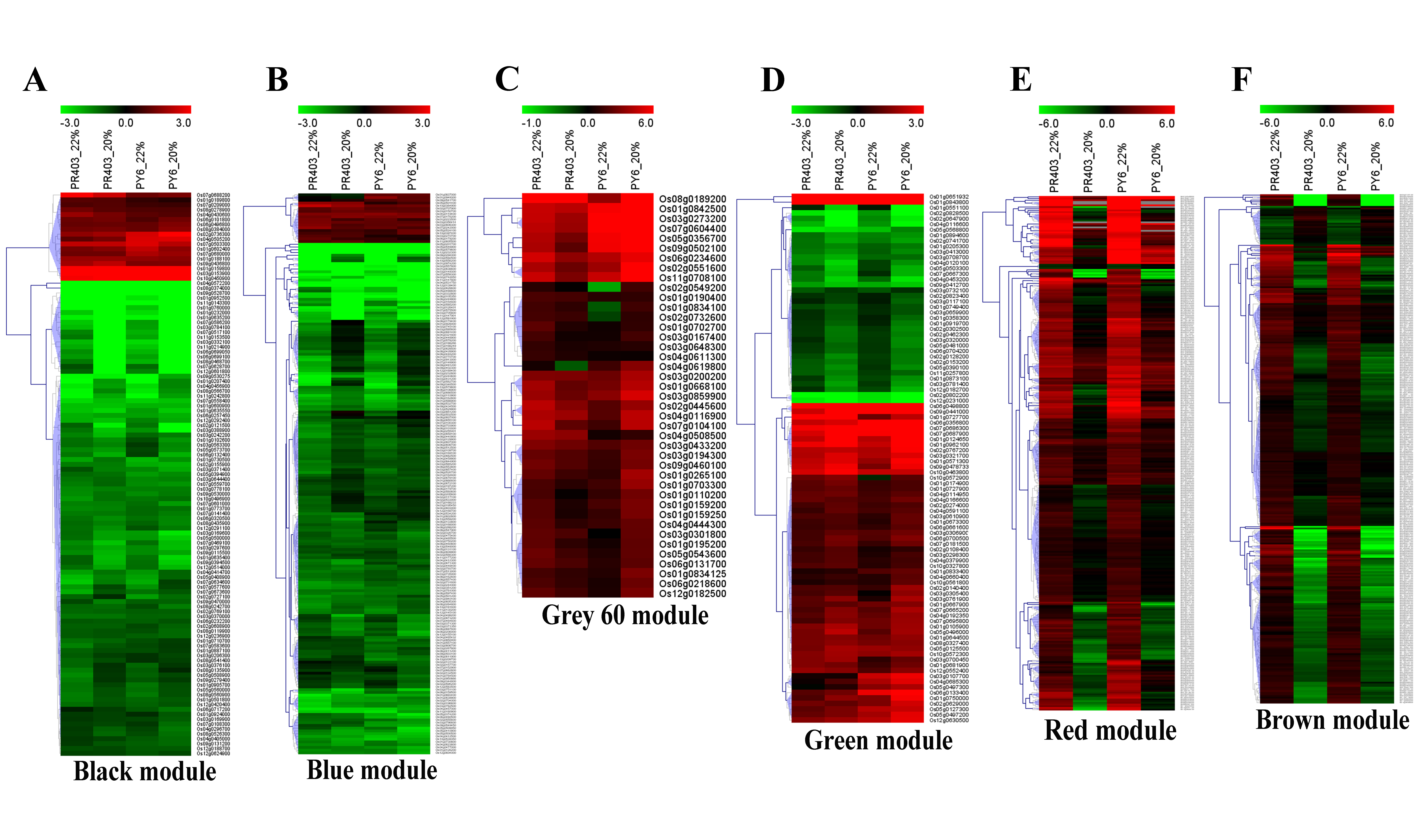

Supplement: Supplementary file 8 — Additional file 8: Figure S8. Heatmap showing that the expression patterns of the hub genes in the modules correlated with H2O2 and MDA accumulation. (A) Module Black. (B) Module Blue. (C) Module Grey60. (D) Module Green. (E) Module Red. (F) Module Brown. All the data used in the analysis were subjected to log2 transformation. [file 12870_2020_2705_MOESM8_ESM.tif]

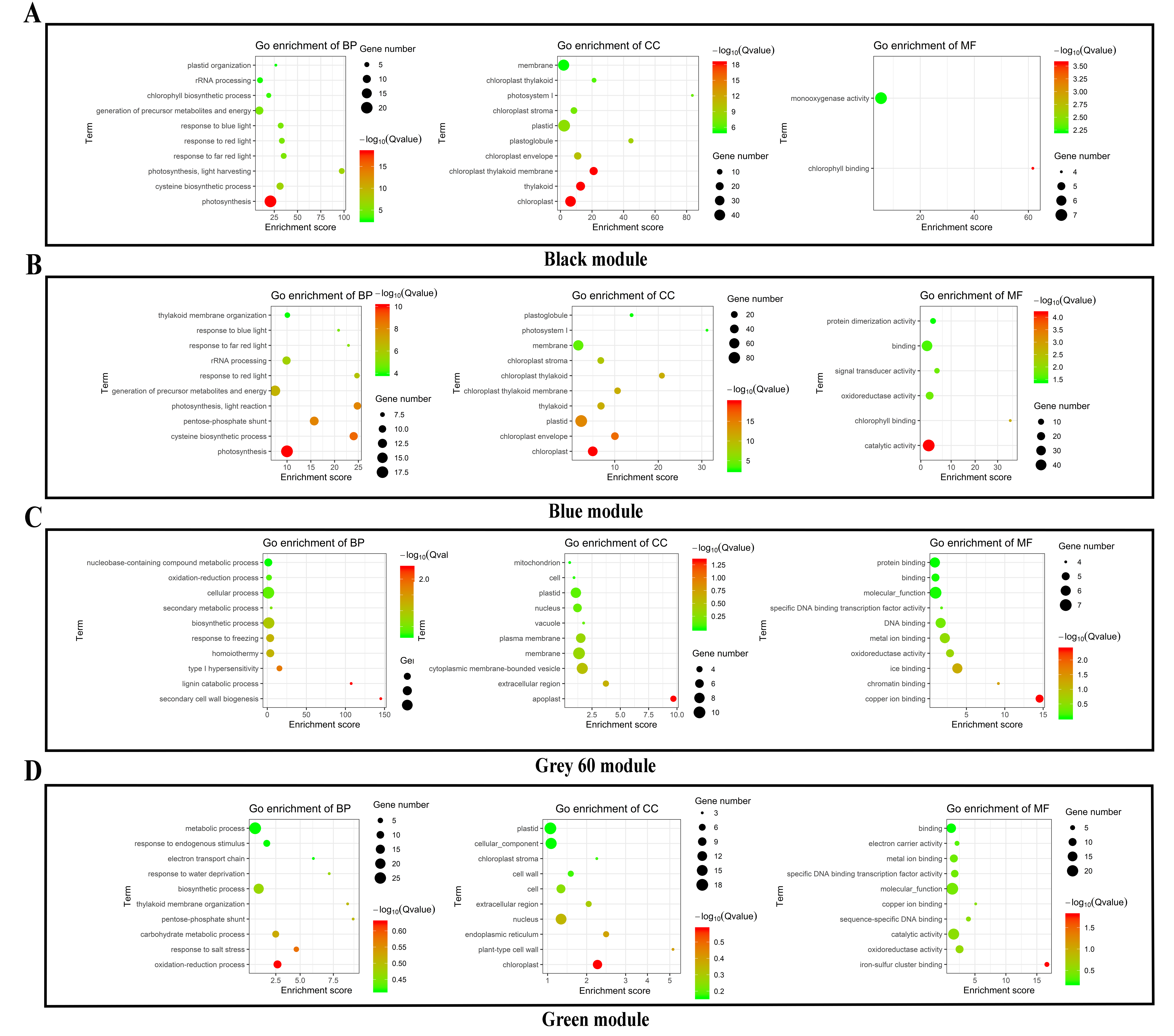

Supplement: Supplementary file 9 — Additional file 9: Figure S9. GO terms overrepresented in the GO enrichment analysis of hub genes in the modules correlated with H2O2 accumulation. The GO terms of biological processes, cellular components, and molecular functions were overrepresented in the modules (A) black, (B) blue, (C) grey60, and (D) green, respectively. Bubble size is proportional to the number of each GO-term, and the color represents the -log10 (Qvalue). [file 12870_2020_2705_MOESM9_ESM.tif]

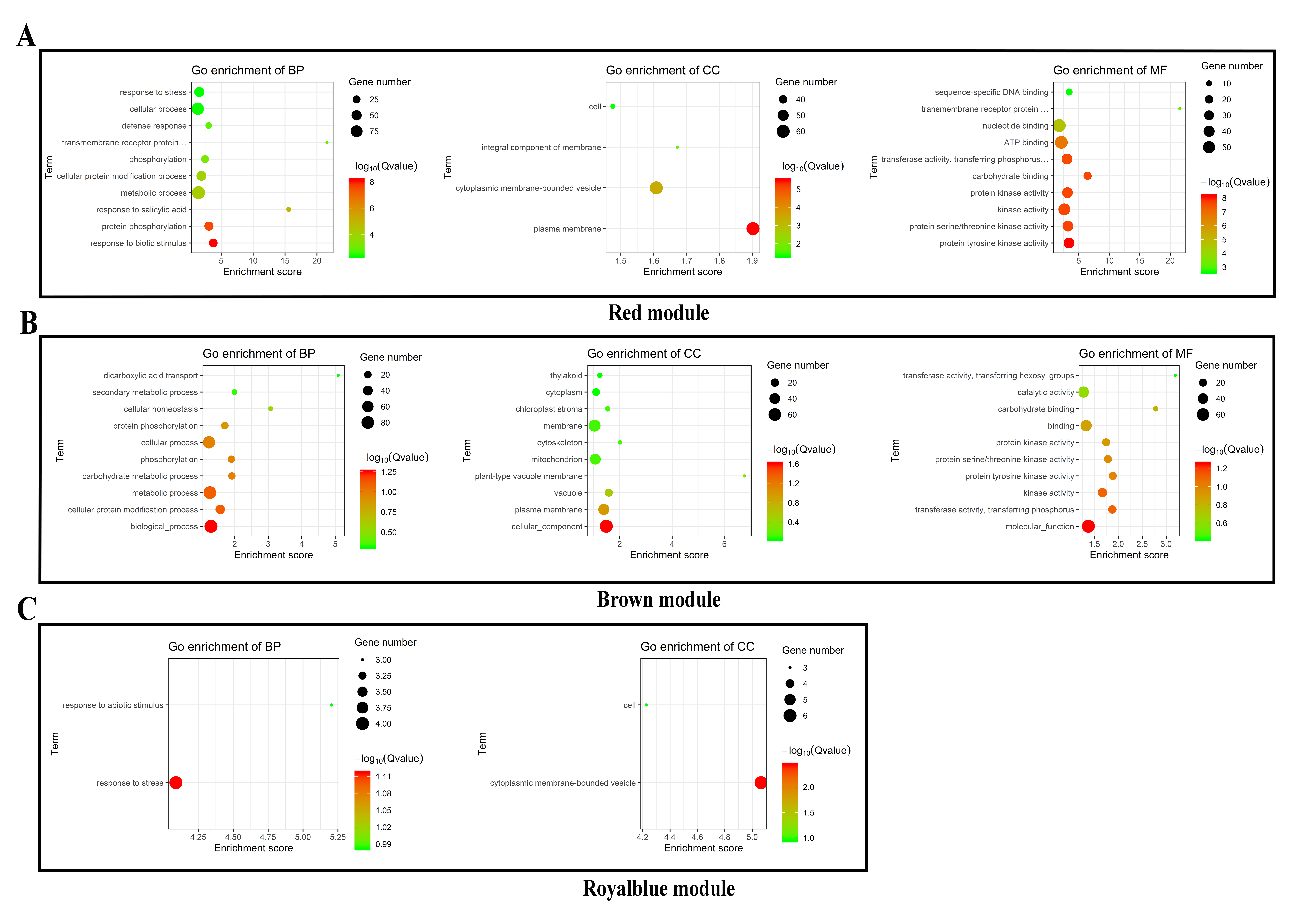

Supplement: Supplementary file 10 — Additional file 10: Figure S10. GO terms overrepresented in the GO enrichment analysis of hub genes in the modules correlated with MDA accumulation. The GO terms of biological processes, cellular components, and molecular functions were overrepresented in the modules (A) red, (B) brown, and (C) royalblue, respectively. Bubble size is proportional to the number of each GO-term, and the color represents the -log10 (Qvalue). [file 12870_2020_2705_MOESM10_ESM.tif]
