# Supplementary material for: Weighted gene coexpression network analysis-based identification of key modules and hub genes associated with drought sensitivity in rice
Source: BMC Plant Biol. 2020 Oct 20;20:478. doi: 10.1186/s12870-020-02705-9 (PMC7576772; doi:10.1186/s12870-020-02705-9)
Supplement: Supplementary file 11 — Additional file 11: Table S1. The primer sequences of genes using for qPCR. [file 12870_2020_2705_MOESM11_ESM.docx]

| **Table S1 The primer sequences of genes using for qPCR** | | |
| --- | --- | --- |
| **Gene ID** | **Forward Primers (5'-3') Reverse Primers (5'-3')** | |
|  |  |  |
| Os02g0115700 | GCCAAGCATGTGAAGAAACTAA | TCTGACATTGTCTGGCCTTATT |
| Os03g0319400 | AGTAGCTCCATCCTTACATGTG | GAGCAGCTATGTACACGAAGAA |
| Os01g0164600 | AAGATGCTTTCTTTGTGAACGG | ATTGACATCCTTTTCTGCCCAT |
| Os04g0610400 | AAACCAACCACCCACCATC | GCCATGTCCGTTCTTGTTC |
| Os01g0289600 | TTGAACTCCTGCAGATTAGTGA | GATAGCCATTGTGCATCGGTAG |
| Os11g0163100 | GAGTATGATGAGTCGGGTCCAG | ACACCAACAATCCCAAACAGAG |
