# Supplementary material for: Weighted gene coexpression network analysis-based identification of key modules and hub genes associated with drought sensitivity in rice
Source: BMC Plant Biol. 2020 Oct 20;20:478. doi: 10.1186/s12870-020-02705-9 (PMC7576772; doi:10.1186/s12870-020-02705-9)
Supplement: Supplementary file 14 — Additional file 14: Table S4. Differentially expressed hub genes identified in module brown. [file 12870_2020_2705_MOESM14_ESM.rtf]

Table S4 Differentially expressed hub genes identified in module brown 

Gene_id	PR403_22a	PR403_20b	PY6_22	PY6_20	Description	GO enrichment	
OS01G0222800	2.84	-3.11	2.28	-4.39	Similar to OSIGBa0145C12.7 protein.	GO:0004674, GO:0005524, GO:0005886, GO:0006468, GO:0016021	
OS11G0569300	1.20	-3.05	2.01	-5.91	Serine/threonine protein kinase domain containing protein.	GO:0004674, GO:0005515, GO:0005524, GO:0006468, GO:0016021	
OS03G0432100	2.07	-0.07	1.44	-1.61	Similar to Pyruvate, phosphate dikinase 2.	GO:0005524, GO:0005737, GO:0006090, GO:0016301, GO:0016310, GO:0046872, GO:0050242	
OS01G0563000	1.53	-6.42	2.00	-5.37	Hypothetical conserved gene.	GO:0000413, GO:0003755, GO:0005528, GO:0005789, GO:0061077	
OS01G0971800	3.24	-3.97	1.52	-6.71	Transcription factor with a GARP DNA-binding domain, Photoperiodic control of flowering time, Clock associated-component	GO:0003677, GO:0005634, GO:0006355, GO:0042753	
OS04G0675400	3.04	-7.53	1.64	-10.21	Similar to Chaperone protein dnaJ.	GO:0009507, GO:0016021	
OS06G0132600	1.00	-2.41	0.79	-3.62	Repair protein Rad1/Rec1 domain containing protein.	GO:0000077, GO:0003684, GO:0006281, GO:0030896	
OS11G0583200	0.63	-2.73	1.65	-1.73	Hypothetical conserved gene.	GO:0005515, GO:0005739, GO:0008270, GO:0016125, GO:0019287, GO:0019288, GO:0034046, GO:0048364, GO:0050790	
OS07G0201500	2.56	-2.09	3.69	-0.16	UDP-glucuronosyl/UDP-glucosyltransferase domain containing protein.	GO:0009813, GO:0043231, GO:0052696, GO:0080043, GO:0080044	
OS09G0545300	3.90	-0.82	3.81	-1.84	SAUR family protein, Negative regulator of auxin synthesis and transport	GO:0005737, GO:0007275, GO:0009734, GO:0046621, GO:2000012	
OS02G0582600	3.37	-2.52	4.57	-1.92	Heavy metal transport/detoxification protein domain containing protein.	GO:0005737, GO:0030001, GO:0046914, GO:0046916	
OS03G0116700	2.70	-0.21	1.71	-1.28	Cyclin-like F-box domain containing protein.	GO:0005515, GO:0009611	
OS11G0209600	1.15	-3.09	1.00	-1.32	Cyclin-like F-box domain containing protein.	GO:0005515	
OS07G0250900	0.73	-3.36	1.61	-1.84	Harpin-induced 1 domain containing protein.	GO:0004871, GO:0006952, GO:0007165, GO:0009506, GO:0016021, GO:0046658	
OS08G0484200	1.36	-2.52	2.07	-1.18	Zinc finger, RING/FYVE/PHD-type domain containing protein.	GO:0005515, GO:0008270, GO:0016021	
OS09G0243200	2.27	-3.24	1.41	-5.35	Zinc finger, RING/FYVE/PHD-type domain containing protein.	GO:0000209, GO:0005515, GO:0008270, GO:0042787, GO:0043161, GO:0061630	
OS10G0395150	1.24	-3.22	1.15	-2.13	Similar to predicted protein.	GO:0003774, GO:0005524, GO:0016459	
OS05G0153400	1.58	-2.94	1.25	-1.73	Pentatricopeptide repeat domain containing protein.	GO:0005515	
 Notes: a represents the samples collected at the 22% of soil moisture content; b represents the samples collected at the 20% of soil moisture content; c the value represents the log2 transformation of the fold change of the expression.
